# A high-resolution data set of fatty acid-binding protein structures. III. Unexpectedly high occurrence of wrong ligands

**DOI:** 10.1107/S2059798325006096

**Published:** 2025-07-28

**Authors:** Andreas Ehler, Christian Bartelmus, Joerg Benz, Inken Plitzko, Markus G. Rudolph

**Affiliations:** a Therapeutic Modalities, Innovation Center Basel F. Hoffmann-La Roche Grenzacherstrasse 124 4070Basel Switzerland; University of Oxford, United Kingdom

**Keywords:** structure-based drug design, fatty acid-binding proteins, ligand binding, unexpected chemical composition

## Abstract

FABP4 has been implicated as a therapeutic target for treating diabetes and atherosclerosis. Structure-based drug design (SBDD) based on initial hits from high-throughput and fragment screens yielded 216 ligand-bound structures of human FABP3, FABP4 and FABP5 isoforms, many of which were at resolutions of better than 1.2 Å. An estimated 15% of the ligands had a different chemical composition to that expected from the starting materials or the final synthesis product, highlighting a potential problem inherent to all SBDD campaigns conducted at lower resolution. Apart from possible human error during compound registration, side reactions such as additions, eliminations, isomerizations, cyclizations and dimerizations were found that led to compounds capable of binding to FABP.

## Abstract 2

Very high-resolution fatty acid-binding protein structures identified a substantial number of ligands that differ strongly from their expected structure. If different ligand chemistry is a potentially overlooked problem in other, lower resolution, crystal structures of protein–ligand complexes, it has repercussions for any study using such structures, particularly when training machine-learning algorithms.

## 1. Introduction

Fatty acid-binding proteins (FABPs) are involved in the uptake, metabolism and intracellular trafficking of fatty acids. Based on epidemiological studies and animal knockout models, the isoforms FABP4 and FABP5 were identified as potential diabetes and atherosclerosis targets (reviewed in Furuhashi & Hotamisligil, 2008 ▸). The aim of this study was to develop a dual FABP4/5 inhibitor and, to this end, high-throughput and fragment screens were conducted with X-ray crystallography as a follow-up method on those ligands that showed appreciable affinity and favorable physicochemical properties. During this exercise, 229 FABP crystal structures from various human and mouse isoforms were determined (Ehler *et al.*, 2025 ▸), many at better than 1.2 Å resolution. 216 of these structures contain a ligand that is well defined by electron density. However, a surprisingly large number of the ligands deviated from expectations. While some ligands suffered from radiation damage at bromine and chlorine sites (Ehler *et al.*, 2025 ▸), sometimes leading to shifts in the ligand pose, other ligands displayed alternate conformations, which may complicate pose-prediction calculations. From a scientific and learning perspective, the most interesting and also largest set of deviating ligands is the ‘what is written on the bottle is not what is in the crystal’ type. 33 out of 216 cases, or 15%, belong to this class of crystal structures. Next to possible human error (mis-labeling), prominent sources of deviations from expectation include side reactions of compounds, such as additions, eliminations, isomerizations, cyclizations and dimerizations. Sometimes these reactions resulted in a complete conversion of the expected compound to a new isobaric molecule. In other cases trace amounts of side products were formed that happened to bind much more tightly to FABP than the parent compound. Similarly, trace amounts of starting materials and degradation products were observed in FABP crystal structures, possibly owing to favorable soaking conditions with up to 60 m*M* ligand in up to 30% DMSO, which the crystals withstood for several hours while retaining their exceptional diffraction properties. The identity of many unexpected ligands could be confirmed by additional NMR and/or mass-spectrometry (MS) measurements, but many ligand identities are evident from the high-resolution electron density alone. Stereochemical considerations such as hydrogen bonds and close-contact analysis of ligands also provided valuable insight into the correct ligand identity. More subtle changes in ligands, including shifts of single atoms, could sometimes be delineated by comparing the anisotropically refined *B* values of wrong and correct ligands.

The high average resolution of the FABP structures and the favorable soaking protocol may have allowed the identification of trace ligands and subtle changes in ligands that might have gone unnoticed in lower resolution structures or with smaller ligand concentrations. In line with this, we also report a few examples at lower resolutions for two other proteins (phosphodiesterase 10 and autotaxin) where the ligand modifications needed to be severe enough to clearly distinguish the binding-competent byproduct from the expected compound. In these projects, each supported by >200 crystal structures, the number of unexpected ligands was far smaller than with FABP, indicating that modified ligands might have gone unnoticed. Overall and in line with previous reports on the high percentage of problematic ligand-containing structures in the PDB (Liebeschuetz *et al.*, 2012 ▸; Deller & Rupp, 2015 ▸), we conclude that the identity of ligands in many other publicly available crystal structures with ambiguous electron density may be unknown or at least the ligand might be chemically different. The presence of such ‘bad’ ligand-containing structures could unnecessarily reduce the value of the PDB as a resource for training ligand-prediction machine-learning (ML) algorithms unless the field agrees on standards for extracting reliable subsets of ligand-bound structures.

## 2. Materials and methods

Here, we only provide details of aspects not described in one of the accompanying publications (Ehler *et al.*, 2025 ▸; Casagrande *et al.*, 2025 ▸). Gene cloning and protein production for both crystallographic and NMR studies are described therein. FABP4_3 is the octuple variant V24L, M41T, I52L, S54T, I105L, V116L, C118L, S125C and FABP4_5 is the octuple variant V24L, V33M, A34G, M41C, S54T, F58L, H94Q, S125C. These variants convert the binding site of human FABP4 into those of the FABP3 and FABP5 isoforms while keeping the FABP4 surface residues for ready crystallization and facile soaking. For crystallographic details and a summary of all crystal structures in the form of an *Excel* table, please see Supplementary Excel File S1. All maps shown in the figures are *F*_o_ − *F*_c_ omit-type (orange) or anomalous maps from SAD phasing with their contour levels and resolution indicated.

The presence of the compound mass was tested by positive-ion electrospray ionization mass spectrometry (ESI-MS) after liquid chromatography (LC). Spectra were recorded on Waters or Agilent LC-MS systems based on single-quadrupole instruments. Separation was achieved on an Acquity BEH C18 2.1 × 50 mm column at 60°C with a gradient of 0.1% formic acid in water against acetonitrile with 0.1% formic acid at a flow rate of 1 mL min^−1^. After a short isocratic hold of 0.5 min at 3% acetonitrile, a linear gradient with a ramp to 99% acetonitrile over about 4.5 min was applied. The ‘UV purity’ was judged from the high-performance LC chromatogram of the compound prior to MS analysis, where the total area of all peaks is set to 100%. If this purity is <90%, the ligand solution was assumed to be a cocktail of several chemical substances. For structure determination by NMR, 1D and 2D spectra were measured on a Bruker Avance 600 spectrometer at 25°C equipped with a 5 mm TCI CryoProbe. Samples were measured in deuterated solvents with the residual solvent peak serving as a reference for chemical shift determination.

## 3. Results and discussion

It is not unusual for solvent-exposed ligands to exhibit some degree of static and dynamic disorder, including alternate conformations of chemical moieties or the ‘absence’ of substituents due to rotational disorder or puckering. FABPs, however, have a buried ligand-binding site that restricts the dynamic disorder of ligands to some extent, and it is these ligands with clear electron density that are discussed here. Among these, arguably the most interesting ligands are those for which the electron density suggests a different chemical composition compared with their assumed structure. About 15% of the FABP4 ligands fall into this category (33 cases; see Supplementary Excel File S1), with 12% (25 cases) where the ligand could be identified unambiguously based on electron density, sometimes aided by NMR and ESI-MS. These numbers are far from anecdotal and point to a systematic reason for the high occurrence. Possible sources for the high rate of unexpected ligands include the high resolution of the data and the high DMSO tolerance of the crystals. The mean and median resolution of the data is 1.21 and 1.12 Å, respectively. Together with the buried ligand-binding site in FABP, the high-resolution data allow the identification of small changes in ligand chemistry, even for peripheral substituents that often display increased flexibility in crystals. Overnight soaks with up to 30% DMSO and 60 m*M* ligand were tolerated by the crystals without loss of diffraction quality. Such favorable conditions may have allowed small impurities to accumulate in the crystal under the assumption that the main component does not bind to the protein. For example, if a small cubic crystal of 100 µm edges containing a 50 kDa protein and an average solvent content of 44% (*V*_M_ = 2.8 Å^3^ Da^−1^) is considered, the volume contains 7 × 10^12^ molecules or 11.8 pmol of protein. If such a crystal is soaked, in a typical scenario, with 2 µL of a 10 m*M* ligand solution that contains a tightly binding impurity, just 590 p.p.m., or 0.06%, of that impurity is needed for equimolarity with the receptor and, given a suitable affinity, to potentially reach 100% occupancy in the crystal. Such low levels of impurity are below the detection limits of several biophysical methods typically used in the quality control of ligands, including HPLC, NMR and mass spectrometry, and thus may escape detection.

A few examples of modified and clearly visible ligands stemming from different possible origins are discussed in the following. Ligands are labeled by their four-character PDB entry codes to avoid an additional labeling scheme. Ligands that did not display clear electron density are not included because dynamic/static disorder cannot easily be distinguished from mixtures of chemical entities which may be present in ligand preparations. If available, analytical results from MS and NMR chemical shift assignment are stated as well.

### 3.1. Possible human error during ligand registration

A trivial source of ‘wrong ligands’ is human error during compound registration, which often involves manual drawing of the chemical structure. Since chemists take great care to register new synthetic compounds correctly in the database, this is a rare source of error. For the registration of purchased compound libraries for screening, a prior annotation error cannot be excluded. Although these sources of potential errors have nothing to do with the chemical synthesis, if the errors are not caught they may lead to delays in the follow-up chemistry of a screening hit.

#### 3.1.1. Wrong heterocycle

**Figure 1:**
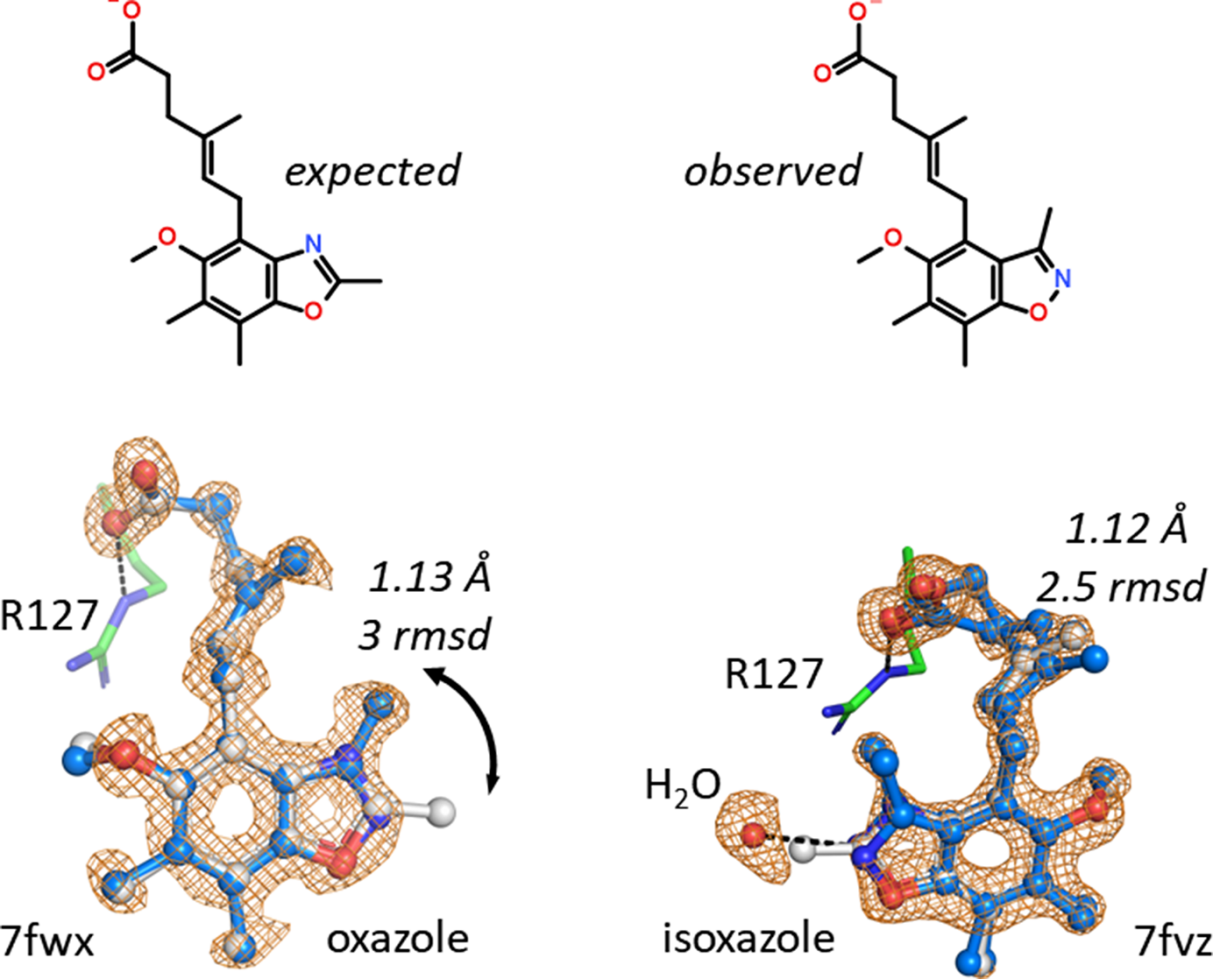
(*E*)-6-(5-Methoxy-2,6,7-trimethyl-1,3-benzoxazol-4-yl)-4-methylhex-4-enoic acid binds to FABP in a similar manner as a normal fatty acid, with its carboxylic group interacting with Arg127. The hFABP4_5 structure 7fwx is to the left and the hFABP4 structure is to the right. In neither case does the methyl group at the benzoxazole moiety fit the electron density (gray ball-and-stick representation), but the density indicates that the methyl group should be shifted by one atom (blue ball-and-stick representation). Structure 7fvz shows a water molecule in contact with the oxazole, indicating that the heterocycle is not a 1,3-oxazole but a 1,2-oxazole or isoxazole.

The crystal structures 7fwx and 7fvz contain the same ligand, an expected 1,3-benzoxazole derivative carrying a methyl group at the 2-position of the oxazole heterocycle (Fig. 1 ▸). The two proteins are hFABP4_5 in space group 18 and hFABP4 in space group 19, respectively, making comparison of the ligand poses interesting. While the carboxylate of the hexenoic acid part of the ligand, just like normal fatty acids, engages in a charged hydrogen-bonding interaction with Arg127, the heterocyclic moiety does not fit the electron density. Both omit electron-density maps suggest that a shift of the methyl group by one atom is necessary. For electronic reasons such a shift is incompatible with a 1,3-oxazole but requires a 1,2-oxazole. Despite the overall high resolutions of 1.13 and 1.12 Å of the diffraction data, the density for the ligands was not sufficiently strong to allow the assignment of elements by anisotropic *B*-value refinement. However, in addition to electronic reasoning, there is a water molecule in structure 7fvz that hydrogen-bonds to the oxazole and requires a heteroatom, not a C atom, at this position. Thus, only 1,2-oxazole (isoxazole) fulfills this requirement.

The molecular mass of the ligand was confirmed by MS, but since the regioisomers are isobaric MS cannot distinguish between them. NMR, on the other hand, clearly supports the presence of the 1,2-oxazole by way of NOEs from the (shifted) methyl group to the methylene group in the hexenoic acid moiety. No synthesis routes exist where both a 2-methyl-1,3-benzoxazole and a 3-methyl-1,2-benzoxazole are products at the same time, making it likely that this compound is a rare case of wrong registration. At lower resolution the error might have gone unnoticed despite a clash between the methyl group and a water in 7fvz.

**Figure 2:**
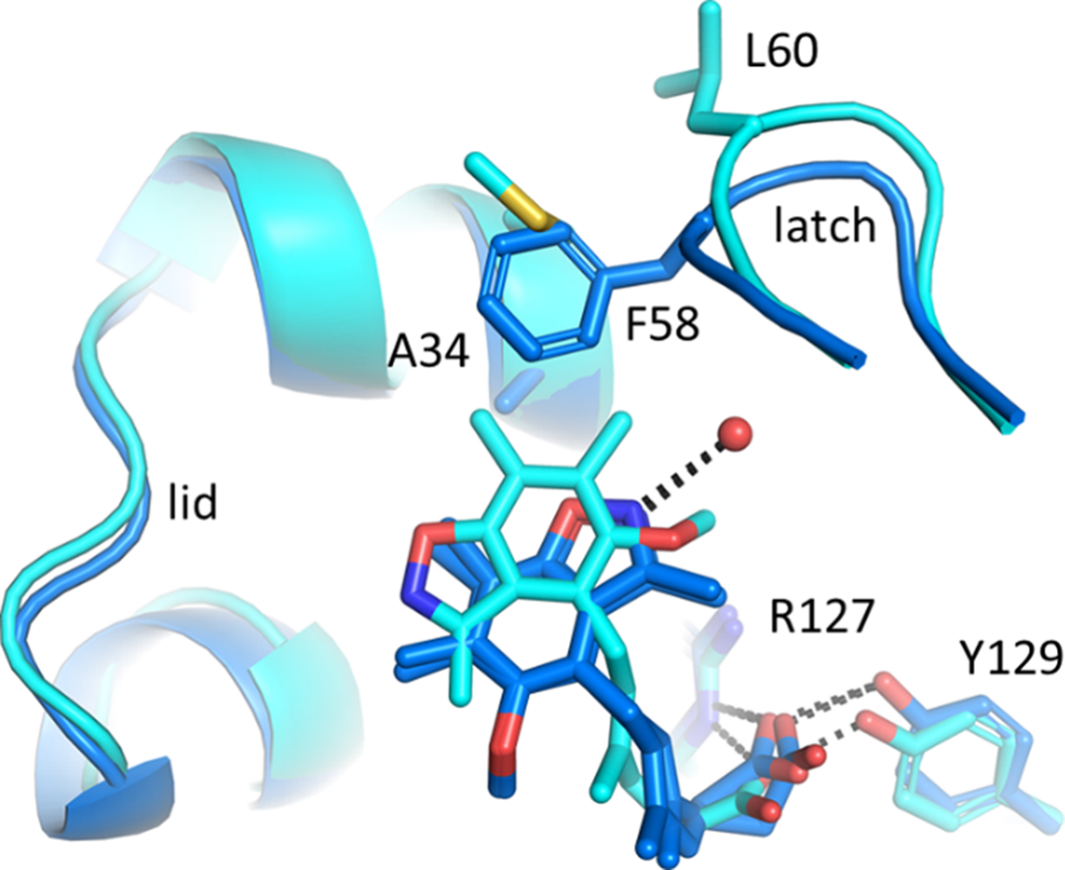
A relative 180° rotation of the benzisoxazole ring is observed in 7fwx (FABP4, dark blue) and 7fvz (FABP4_5, cyan). Position 34 is an alanine in FABP4 but is a glycine in FABP5. The presence of the alanine methyl side chain is incompatible with a methyl group on the heterocycle of the ligand, leading to a flip of the entire group.

The 180° flip of the heterocyclic ligand part in 7fwx (FABP4_5) compared with 7fvz (FABP4) is explained by the presence of Ala34 in the lid region of FABP4, which is a glycine in the FABP5 surrogate FABP4_5 (Fig. 2 ▸). The methyl side chain of Ala34 in FABP4 does not allow approach of the heterocycle, keeping it in a rotamer where the two methyl groups at the ligand are distant from Ala34. This allows the recruitment of a water molecule by the ligand in 7fwx and also for the side chain of Phe58 from the latch region to flip in, packing on top of Ala34. By contrast, the presence of Gly33 in FABP5 does allow approach of the ligand methyl groups to the lid region, at the expense of the latch residue Leu57 in FABP5 flipping away from the lid region. The subtle differential binding of the same ligand to different FABP isoforms has direct consequences for the position of the latch, and thus may have biological effects.

#### 3.1.2. Wrong assignment of chiral centers in enantiomers and diastereomers

**Figure 3:**
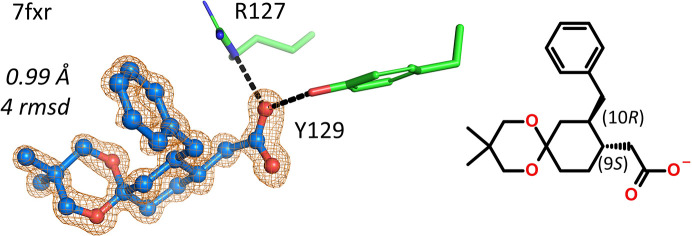
Electron density-based assignment of the correct enantiomer of an enantiopure substance of previously unknown absolute configuration.

Compounds containing stereocenters of unknown geometry are registered as a mixture in our database, whereas if their chirality is known they are registered as individual enantiomers/diastereomers. A gray zone contains compounds that are known to be pure enantiomers but for which the chirality of their stereocenters is not yet defined, for example in separated racemic mixtures for which it is not known which part is (*S*) and which part is (*R*). In such cases, the electron density is usually the deciding factor for final assignment of the correct enantiomer. The 0.99 Å resolution hFABP4 structure 7fxr contains one of the enantiomers of 2-[10-benzyl-3,3-dimethyl-1,5-dioxaspiro[5.5]undecan-9-yl]acetic acid, and the omit electron density clearly established that it was the (9*S*,10*R*) enantiomer that bound to FABP4 (Fig. 3 ▸).

**Figure 4:**
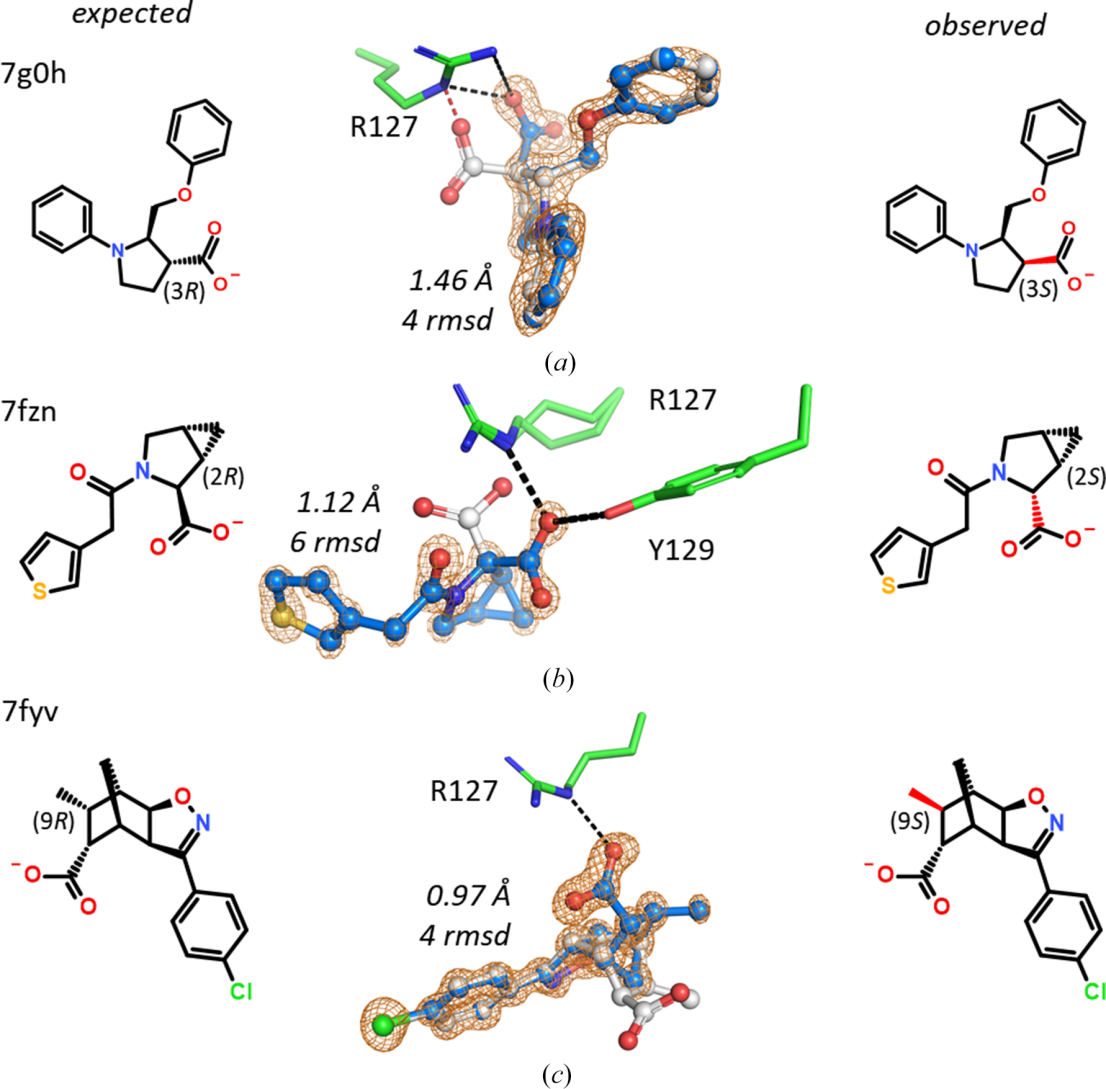
Electron density-based assignment of the correct diastereomer. The bond in question is marked in red. (*a*) Instead of (2*R*,3*R*), this 2-(phenoxymethyl)-1-phenyl-pyrrolidine-3-carboxylic acid is (2*R*,3*S*). (*b*) Instead of (1*S*,2*S*,5*R*), this 3-[2-(3-thienyl)acetyl]-3-azabicyclo[3.1.0]hexane-2-carboxylic acid is (1*S*,2*R*,5*R*). (*c*) Only a single one of the six stereocenters in this compound was found to be wrong. Instead of (1*S*,2*R*,6*R*,7*S*,8*R*,9*R*), the 5-(4-chlorophenyl)-9-methyl-3-oxa-4-azatricyclo[5.2.1.0^2,6^]dec-4-ene-8-carboxylic acid is (1*S*,2*R*,6*R*,7*S*,8*R*,9*S*). Unfortunately, no NMR or MS analysis was performed for any of these three compounds.

For pure diastereomers, the assignment of chiral centers must be explicit, with the only uncertainty being which of the two enantiomers is present, *i.e.* all centers must be defined correctly as (*S*)/(*R*) or alternatively the compound is the mirror image with all of these centers inverted. However, in the FABP4 data set there are three examples (7fwx, 7fznand 7fyv) where the ligand turned out to be a different diastereomer (*i.e.* not the enantiomer; Fig. 4 ▸). Errors like this are likely not to be a problem if the chemical synthesis includes the stereocenters in the starting materials, but may become problematic if the chiral moiety is synthesized *de novo*.

#### 3.1.3. No explanation

**Figure 5:**
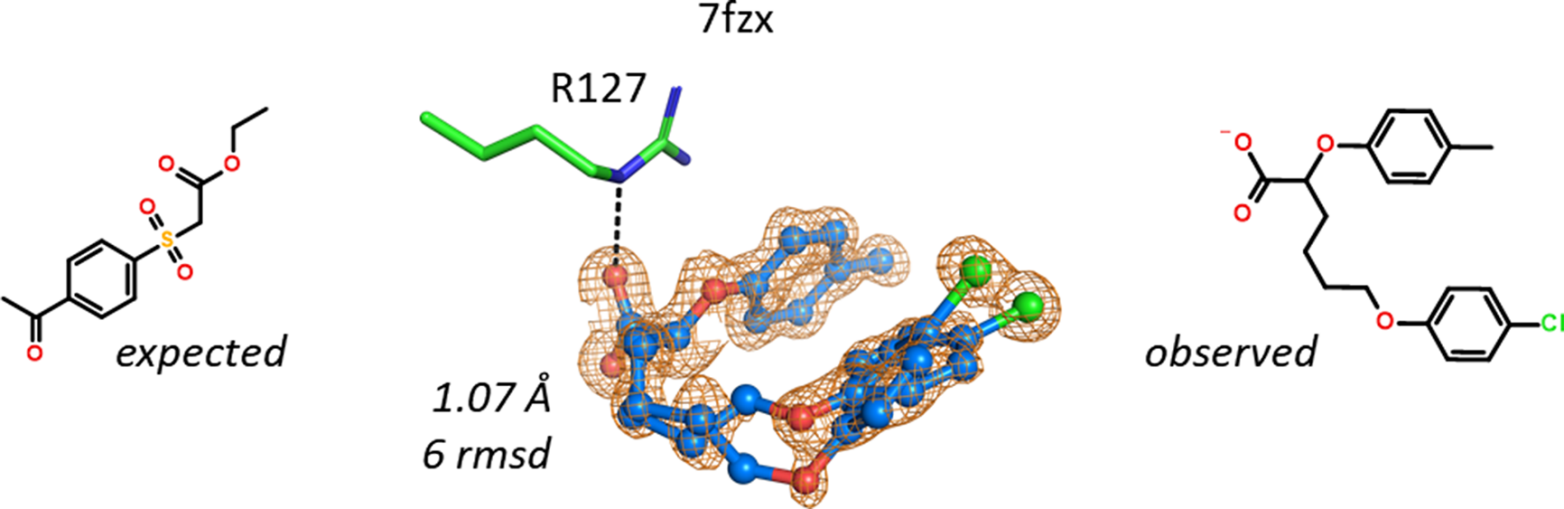
The sulfonyl acetate expected is not even remotely similar to the observed 6-(4-chlorophenoxy)-2-(4-methylphenoxy)hexanoate. Two conformations are observed in the crystal with different placement of the chlorophenoxy group.

In one case the chemical structures of the expected and observed ligands in the crystal structure were so different that this example stands out as a curiosity. In the 1.07 Å resolution hFABP4 structure 7fzx, a methylphenoxy hexanoate was observed instead of a sulfonyl acetate (Fig. 5 ▸). Both molecules are present in the compound library, but their registration numbers are unrelated, so an accidental swap of digits can be ruled out. The new structure is well defined by electron density, despite the presence of alternate conformations, and it was also confirmed by NMR.

### 3.2. Covalent modification by ring opening

**Figure 6:**
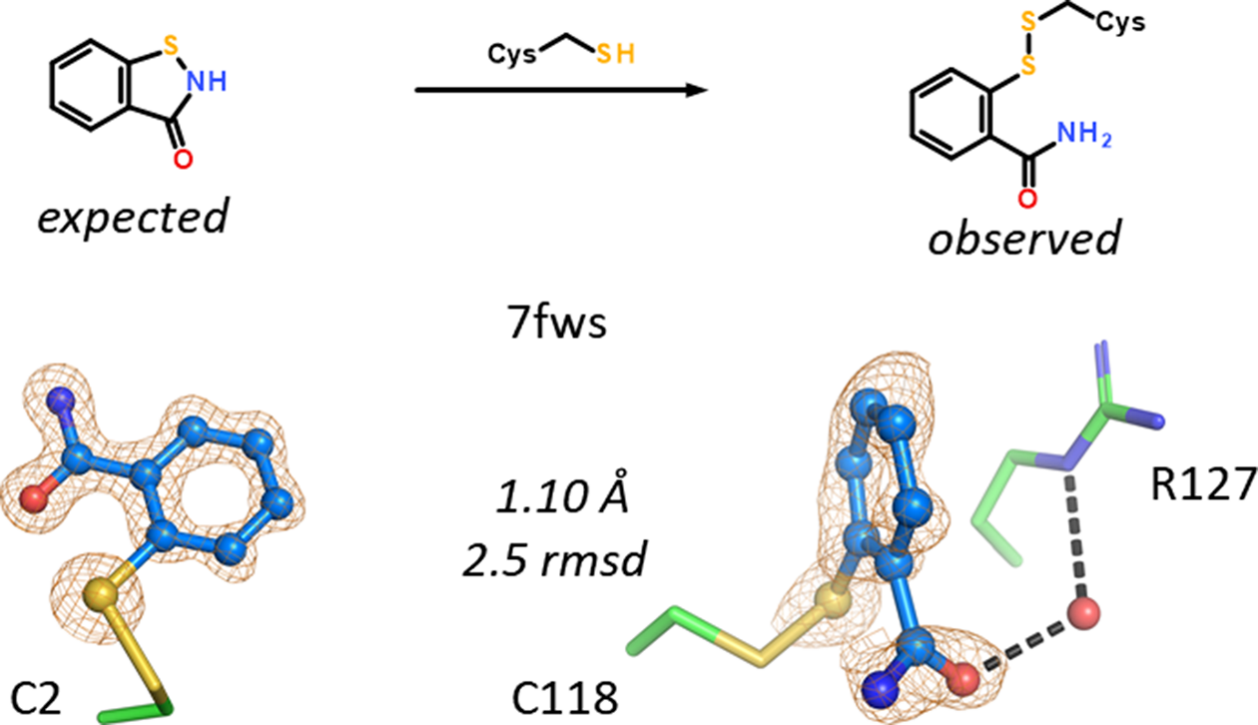
1,2-Benzothiazol-3-one is an electrophile that may be ring-opened by cysteines to form a disulfide with 2-thiobenzamide. Two cysteines, Cys2 and Cys118, are modified in hFABP4 structure 7fws.

The covalent modification of the protein by a sufficiently reactive compound can probably be considered a trivial case of unexpected ligand occurrence. A single example is present in the FABP data set: 7fws is hFABP4 in complex with the fragment 1,2-benzothiazol-3-one determined at 1.10 Å resolution. The benzothiazol-3-one ring is opened by the nucleophilic attack of cysteine C^γ^ atoms at the S atom of the thiazol-3-one. Two sites in hFABP4 are modified: Cys118 in the ligand-binding site and Cys2 near the N-terminus. The result in both cases is a disulfide bond between cysteine and 2-thiobenzamide, *i.e.* the amide of thiosalicylic acid (Fig. 6 ▸). Of note, modification at Cys118 displaces the conserved side chain of Phe17, which in all other FABP structures in complex with any other ligand does not shift appreciably.

### 3.3. Intramolecular condensation reaction leading to ring closure

**Figure 7:**
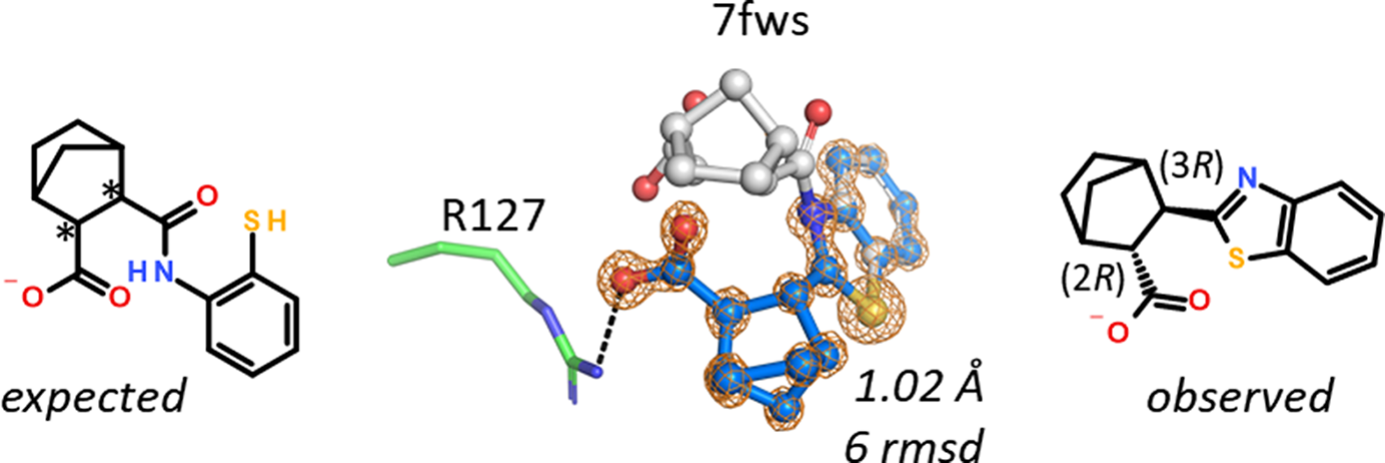
Intramolecular condensation of a 2-sulfanylphenyl-carbamoyl group to a benzothiazole in 7fws. Of the four diastereomers possible in principle, the crystal selected the (2*R*,3*R*) isomer.

hFABP4 structure 7g1v, determined at 1.02 Å resolution, contains a norbornane-2-carboxylic acid derivative. The ligand has two stereocenters but was registered in our database without stereochemical information, which points to a mixture of up to four isomers. Upon fitting of the 2-sulfanylphenyl-carbamoyl substituent located *ortho* to the acid into the omit map, the remainder of the map could not be interpreted with ligand atoms unless severe stereochemical clashes were permitted. The electron density is of high enough quality to visualize individual atoms (Fig. 7 ▸), and it was immediately clear that a five-membered cyclic arrangement is present fused to the benzene moiety. Fusion of the 2-sulfanylphenyl-carbamoyl group to a benzothiazole allowed the norbornyl carboxylic acid to bind to Arg127. Since the condensation leads to intramolecular ring closure with the elimination of a water molecule, the mass difference is amenable to MS analysis, which indeed confirmed the new molecular mass.

### 3.4. Side reactions

#### 3.4.1. Different substituents present than expected

**Figure 8:**
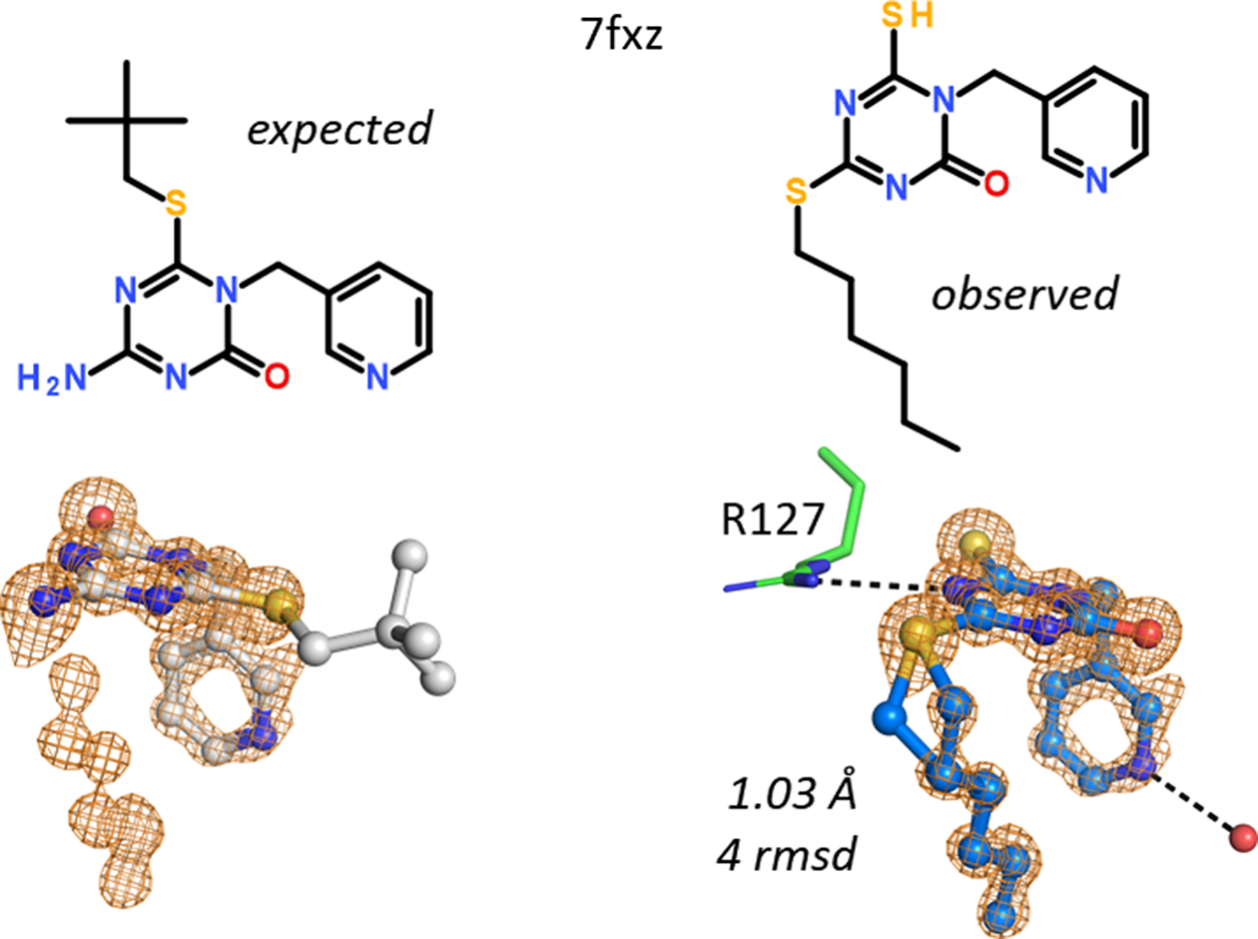
Instead of a *tert*-butyl substituent, this compound includes an *n*-hexyl located at a second S atom on the triazine core.

In hFABP4 structure 7fxz, the nature and position of an aliphatic substituent was different to expectations. Instead of a *tert*-butyl thioether bound to a 1,3,5-triazin-2-one core, an *n*-hexyl substituent was found bound at an additional S atom at a different position of the triazine (Fig. 8 ▸). Due to its large atomic mass, the additional S atom was easily detected from the omit map. A water molecule helped in unambiguous placement of the pyridyl substituent by way of hydrogen-bond considerations. The molecular mass and the chemical structure of the observed compound were later confirmed by MS and NMR, respectively. The compound was added to our library in 1997 and it is currently unclear at which step during the synthesis this modification could have occurred.

#### 3.4.2. Acyl shift

**Figure 9:**
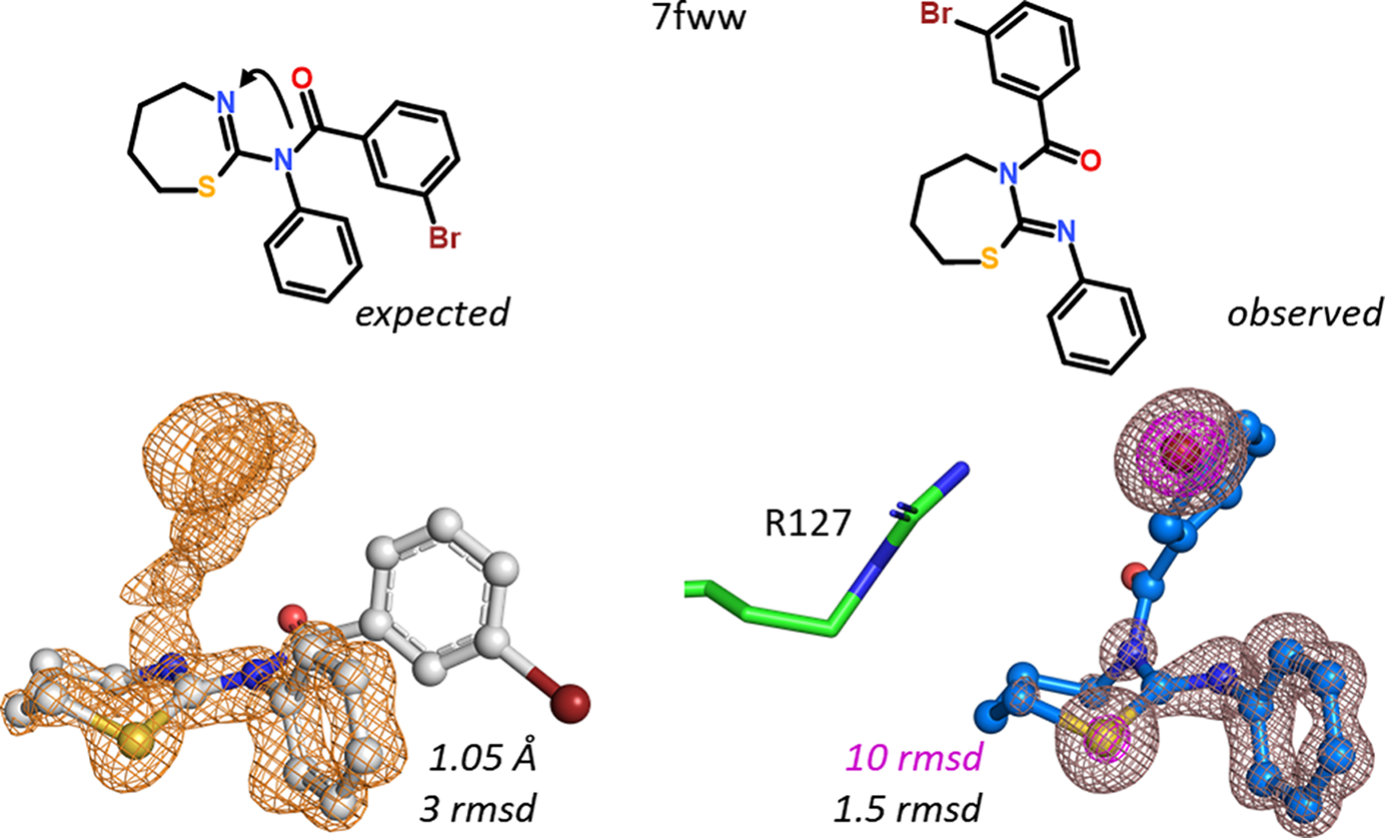
N–N acyl shift of a 3-bromophenyl group in a thiazepine/thiazepane system. The omit map contoured at 3 r.m.s.d. is at the bottom left. The experimental SAD map on the bottom right is contoured at 1.5 r.m.s.d. (brown) and 10 r.m.s.d. (magenta), showing the positions of the S and Br atoms in the ligand. While no NMR data are available on this compound, and MS cannot distinguish between the isobaric regioisomers, the density maps are straightforward to interpret. Arg127 is included for reference; no direct hydrogen bonds between ligand and FABP4 are present in the structure.

A peculiar ‘shift’ of a 3-bromophenyl group from one nitrogen to another is visible in hFABP4 structure 7fww. The original assumption was a thiazepine benzamide derivative, but the omit electron density showed that the 3-bromophenyl group resided on the N atom in the seven-membered heterocycle, making this compound a thiazepane. An independent proof of the nature of this compound came from SAD phasing (see also Ehler *et al.*, 2025 ▸). Data were collected at 0.7 Å wavelength, which yields a calculated *f*′′ of 2.4 e^−^ for bromine. The single Br atom in the ligand delivered enough anomalous signal to phase the structure using *SHELXD*/*E* (Fig. 9 ▸). Interestingly, this compound does not contain an acidic moiety and there are no direct hydrogen bonds present between the ligand and hFABP4.

### 3.5. Possible leftover starting material: missing substituents

If electron density defines a ligand as smaller than anticipated, possible reasons include rotational disorder of parts of the ligand, decomposition of the ligand upon storage or during crystallization, or the presence of trace amounts of starting materials of the synthesis. In the following two sections, only such cases are presented where rotational disorder could be excluded. A distinction between decomposition and the presence of starting material is not always possible, especially when decomposition could have happened by hydrolysis and the synthesis was performed using a condensation reaction (see Section 3.6).

#### 3.5.1. An unusual acidic group instead of a heterocycle

**Figure 10:**
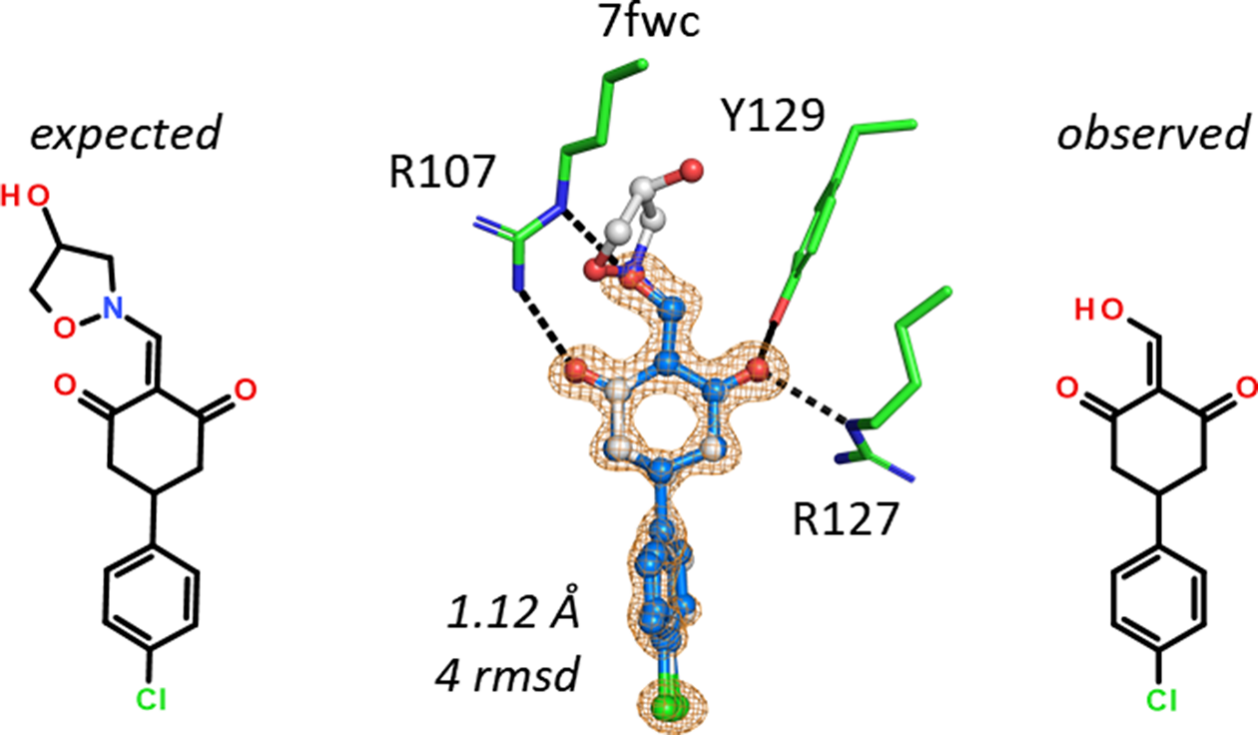
The anticipated hydroxyisoxazolidine substituent (gray) in 7fwc would not fit into the binding pocket of FABP4. Instead, a smaller compound that was identified as 5-(4-chlorophenyl)-2-(hydroxy­methylene)cyclohexane-1,3-dione is bound to Arg107.

A cyclohexane-1,3-dione carrying a hydroxyisoxazolidine substituent was expected in hFABP4 structure 7fwc. The 1.12 Å resolution maps did not support the hydroxy­isoxazolidine, which would not fit into the binding pocket. Instead, only two extra nonlinear atoms in plane with the 1,3-dione are visible in the omit map (Fig. 10 ▸). Arg107 forms two hydrogen bonds: one to a keto group of the 1,3-dione and the other to the terminal atom of the unknown group, which is therefore likely to be an O atom acting as a hydrogen-bond acceptor. An amino group is not an alternative as the analogous enamine is prone to hydrolysis. This compound could have served as a starting material for the target hydroxy­isoxazolidine. The molecular mass of the compound suggested from the omit map and the hydrogen-bonding pattern was indeed detected in the original ligand solution by mass spectrometry, among other impurities. Interestingly, this compound does not have a very acidic group such as a carbonic acid. The calculated p*K*_a_ value for the terminal enol hydroxyl group is 6.2, hence the ligand may bind as an enolate to Arg107. The residues Arg127/Tyr129 that are most frequently observed to bind to acidic groups in FABP ligands bind to the second keto group of the 1,3-dione.

#### 3.5.2. A missing ethyl group: incomplete alkylation reaction

**Figure 11:**
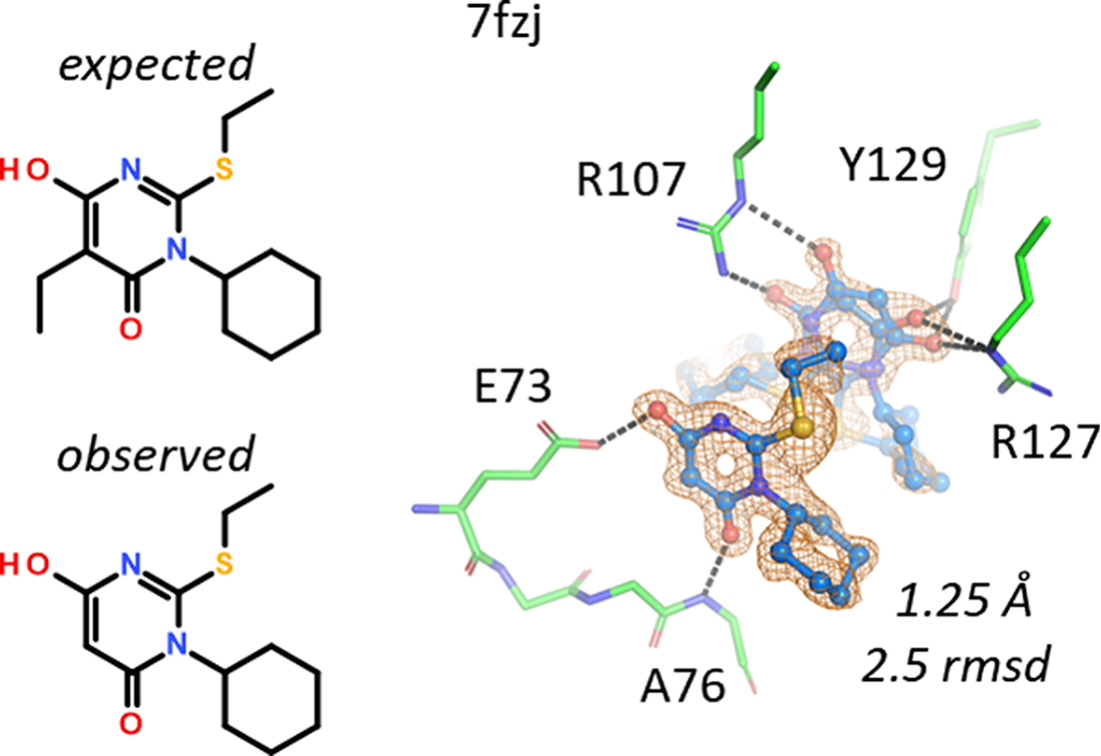
An ethyl group is missing in the hydroxypyrimidin-4-one ligand in 7fzj. The binding site has no space for the anticipated ethyl group but hosts 3-cyclohexyl-2-ethylsulfanyl-6-hydroxy-pyrimidin-4-one. There are two molecules of the ligand in the binding site, one of which was built in two alternate conformations. The OH group of the ligand has a calculated p*K*_a_ value of 7.4.

A 6-hydroxypyrimidin-4-one with an ethyl group at the 5-position was expected for structure 7fzj. The 1.25 Å resolution omit map showed the presence of two ligands in the binding site, one of which was disordered and had to be built in two conformations (Fig. 11 ▸). For none of the ligands was there space for the 5-ethyl group, indicating that starting material without the ethyl group was still present in the ligand solution. MS and NMR analyses confirmed the presence of both compounds, with and without the 5-ethyl group, so the crystal selected the best-fitting molecule. The disordered copy of the ligand (in the background of Fig. 11 ▸) engages in the usual interactions with Arg107, Arg127 and Tyr129. The second, better-ordered copy of the ligand stacks on top of the first ligand and hydrogen-bonds to the side chain of Glu73 and the main-chain NH group of Ala76. The interaction of the ligand with Glu73 also indicates that the 6-position of the ligand indeed carries a hydroxyl group, fixing the tautomer of the ligand. The calculated p*K*_a_ value of this OH group is near neutrality, such that the enol can act as a hydrogen-bond donor to the deprotonated Glu73 in this structure. Thus, 7fzj appears to be one of the rare cases where assignment of the tautomer based on hydrogen-bonding networks is straightforward (Borbulevych *et al.*, 2016 ▸; Bax *et al.*, 2017 ▸).

### 3.6. Decomposition by hydrolysis or leftover starting material

#### 3.6.1. Ring opening or failed ring closure

**Figure 12:**
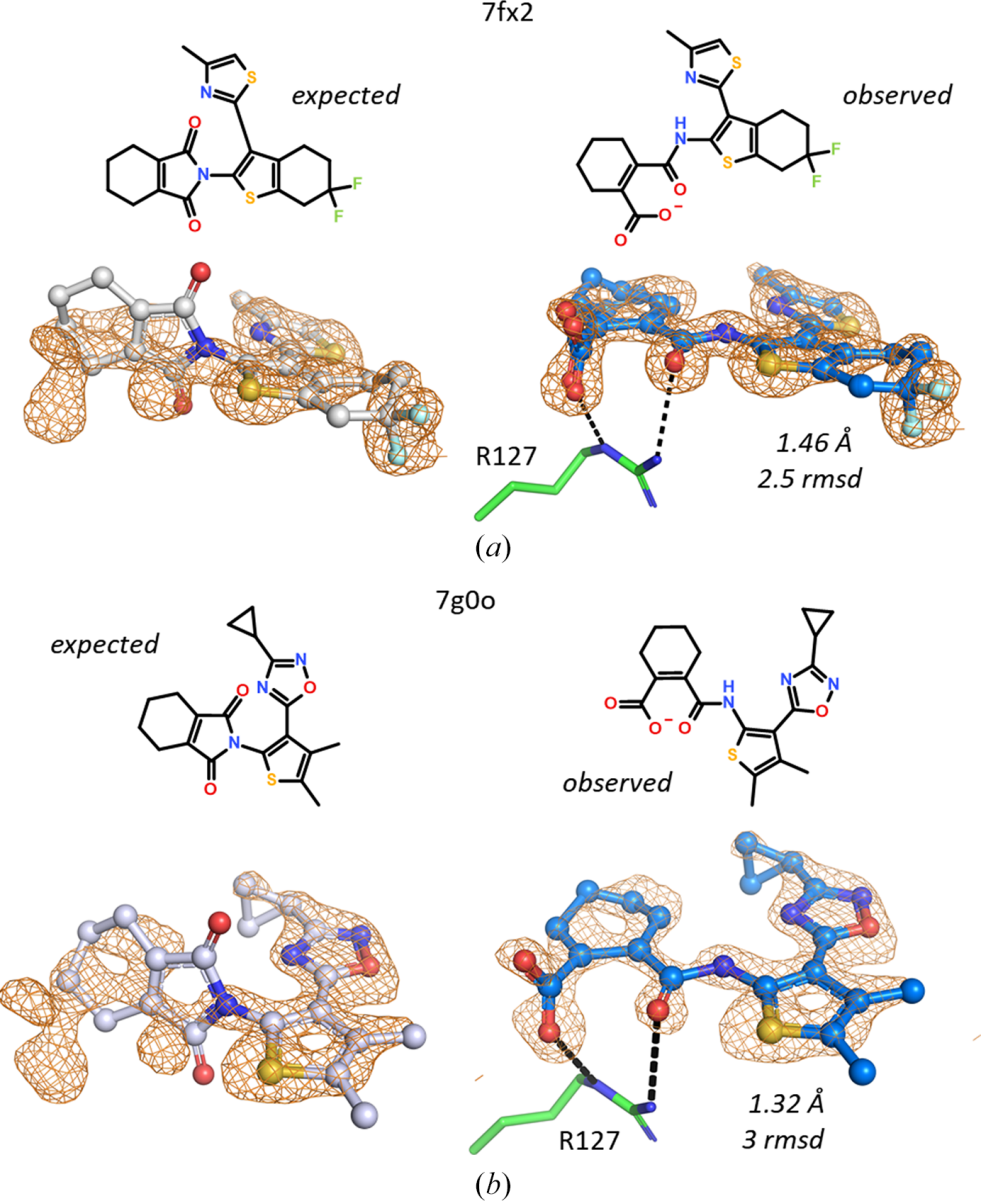
Incomplete ring closure or hydrolysis. (*a*) 7fx2. (*b*) 7g0o. Omit maps are shown in both the left and right panels for clarity. The tetrahydro­isoindole-1,3-dione ligands on the left do not fit the omit maps contoured at 2.5 r.m.s.d. (7fx2) or 3 r.m.s.d. (7g0o). In both cases the open form of the imide is a cyclohexene-1-carboxylic acid that favorably binds to Arg127.

The FABP data set contains two closely related N-substituted pyrrole-1,3-dione ligands that bind in an open form to hFABP4_5. In the 1.47 Å resolution structure 7fx2, even at the comparatively lower resolution compared with other FABP structures, the compound clearly does not fit the omit electron density unless the pyrrole-1,3-dione ring is opened (Fig. 12 ▸). The same is true for the 1.32 Å resolution structure 7g0o. The presence of the open form as an impurity in both ligand preparations was confirmed by MS.

Ring opening is not uncommon and has also been found in 2-methylbenzoxazoles, which can hydrolytically open in a presumably acid-catalyzed reaction to the hydroxyphenyl acetamide, a reaction that may occur with hydrochloride salts even in the solid state (Davis & Erlanson, 2013 ▸). In hindsight, it cannot be decided whether the target tetrahydroisoindole-1,3-dione compound suffered hydrolysis or whether the synthesis was incomplete.

#### 3.6.2. Hydrolysis of esters or incomplete esterification

**Figure 13:**
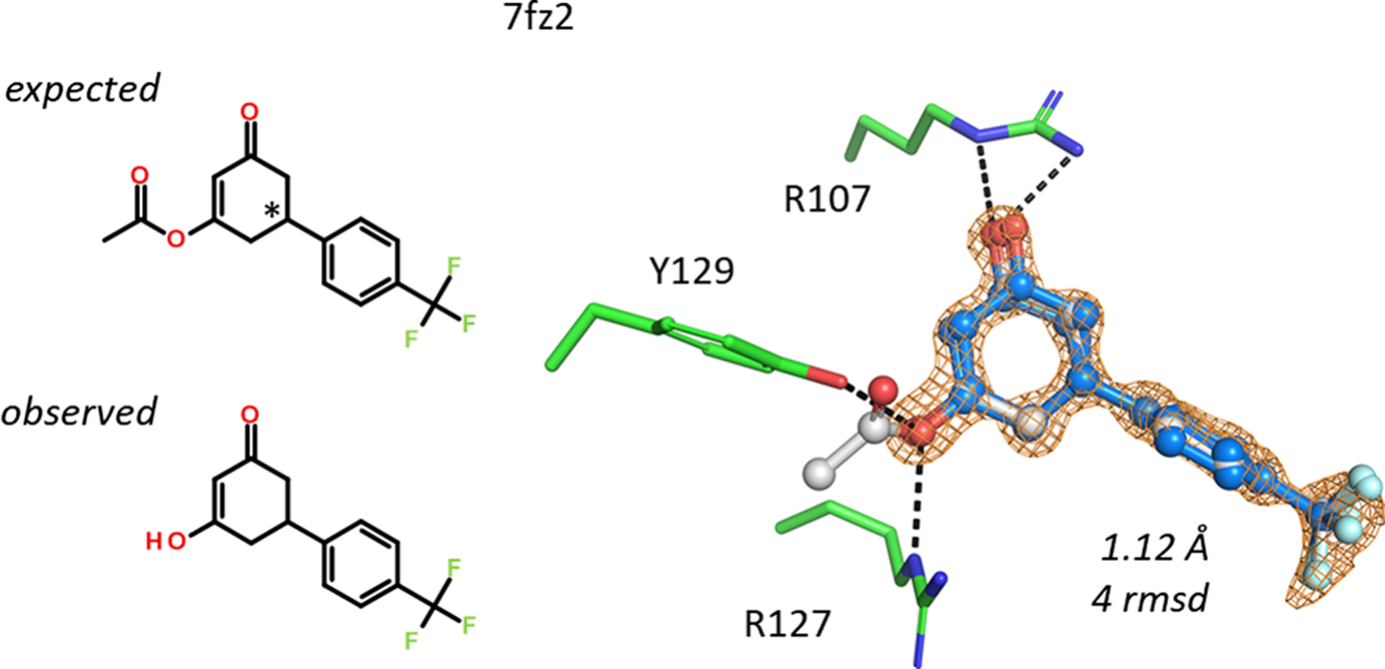
The ethyl ester in this ligand does not fit into the binding site in 7fz2. Only the free hydroxyl form was observed in the electron-density map.

The ligand [3-oxo-5-[4-(trifluoromethyl)phenyl]cyclohexen-1-yl] acetate is an ester of the enol tautomer of cyclohexane-1,3-dione. This molecule is chiral, but once the ethyl ester is hydrolyzed (or not formed) an achiral molecule binds to hFABP4 in 7fz2 (Fig. 13 ▸). The calculated p*K*_a_ value of the enol is 4.6, indicating that the enolate might be present in the structure. Both O atoms of the cyclohexane-1,3-dione or tautomers are engaged in hydrogen bonds to the nearby Arg107 and Arg127, which leaves no space for an ester group. Although no analytical data are available for this compound, these considerations are sufficient to assign the correct chemistry to the ligand. The ligand itself appears to be mobile, and two conformations were built with a different pucker at the C atom where the trifluoromethylphenyl group is attached and slightly different rotations of the CF_3_ group.

**Figure 14:**
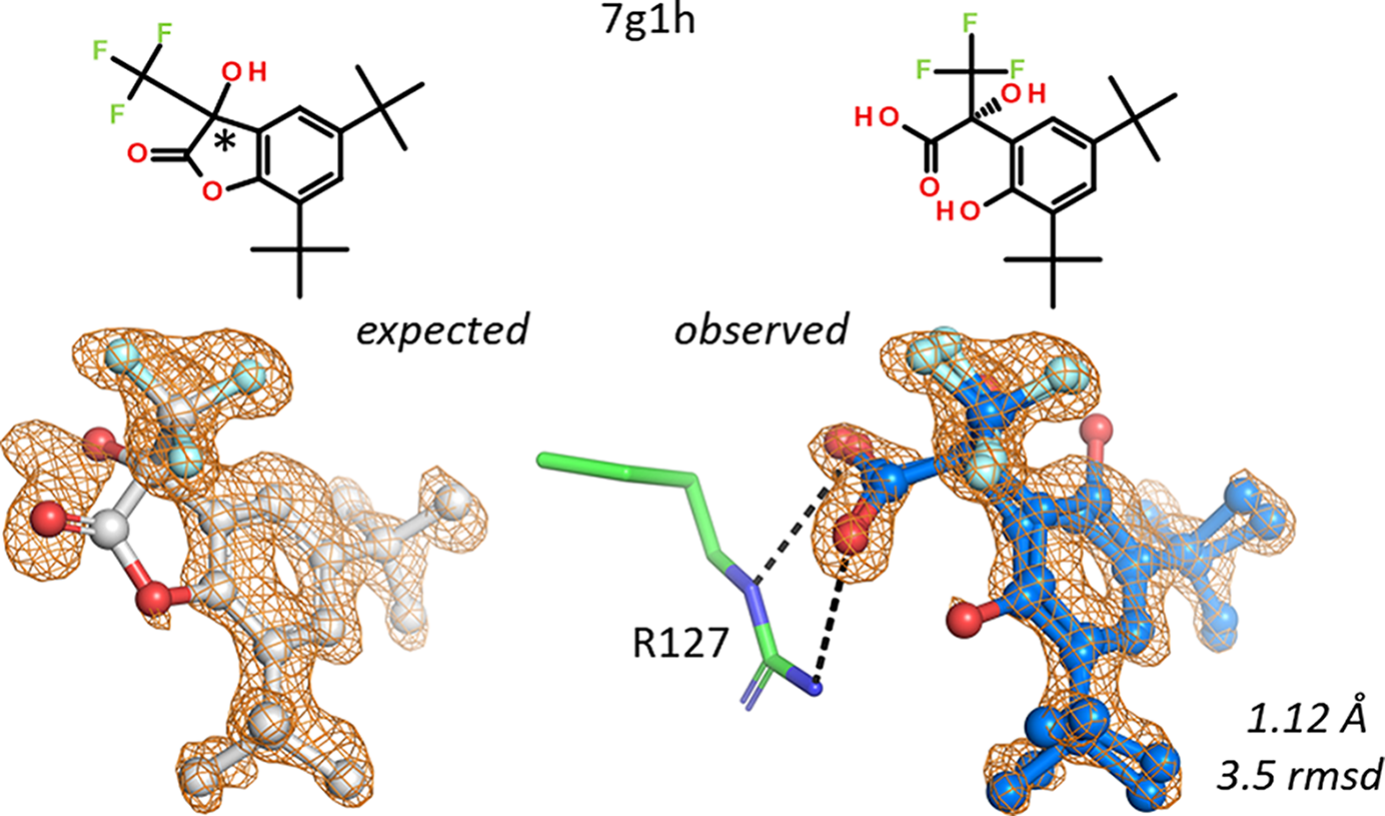
The expected lactone in the ligand does not bind to FABP4, but the open hydroxy acid does, contacting Arg127 and Tyr128 (not shown). Two alternate conformations of the ligand are supported by electron density.

A second example is an expected lactone-containing ligand in hFABP4 structure 7g1h. Only the open form of the lactone, the hydroxy acid, can bind to FABP4 in the usual way where the carboxylate contacts an arginine, here Arg127 (Fig. 14 ▸). The ligand is chiral and was soaked as a racemic mixture, from which the crystal selected the (*R*)-enantiomer. Two alternate conformations of the ligand needed building that placed the phenolic hydroxyl group on either side of the benzene ring. Both orientations allow intramolecular hydrogen bonding of the ligand, in one case with the carboxylic acid and in the other case with the tertiary hydroxyl group of the hydroxy acid.

#### 3.6.3. Decomposition of a 3-keto-sulfoxide

**Figure 15:**
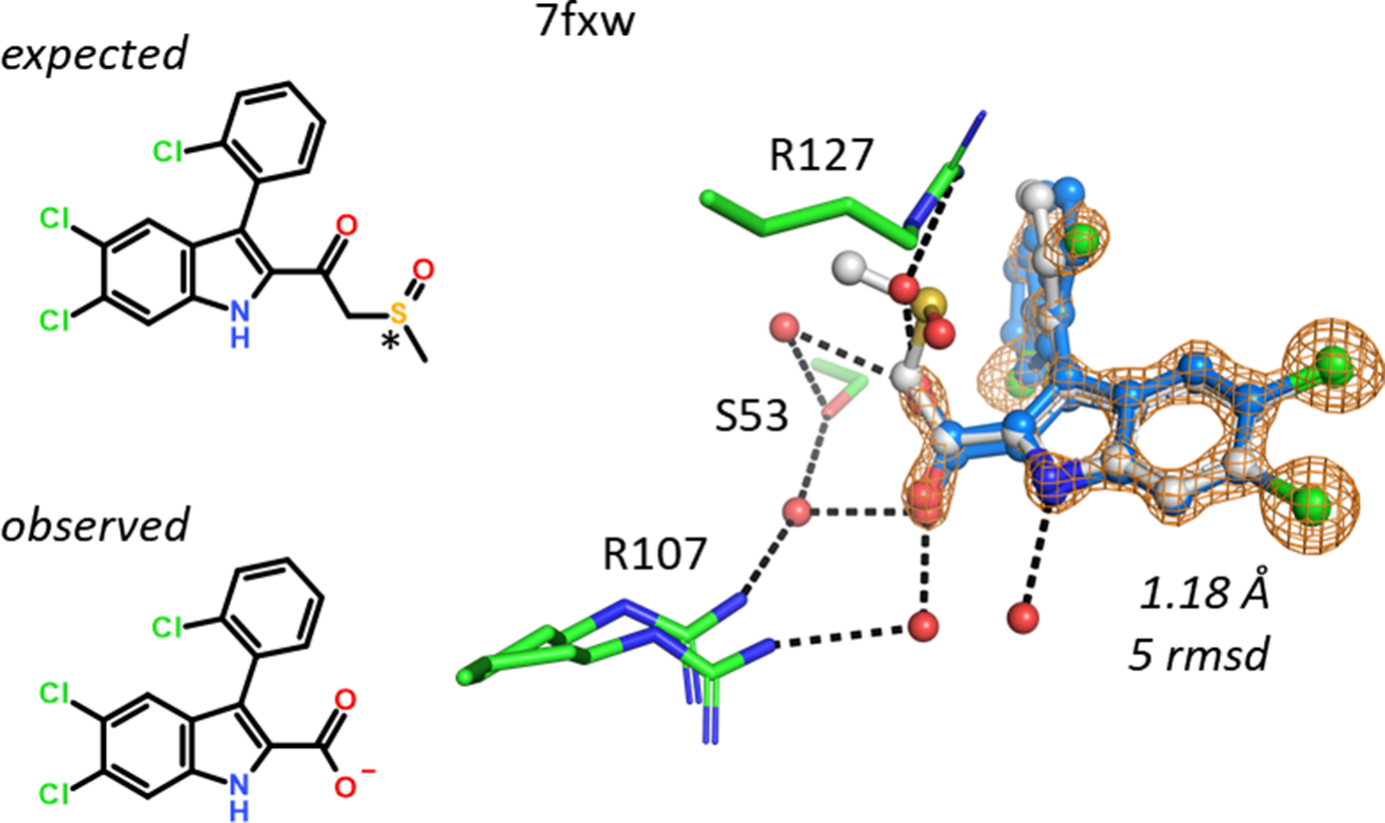
The sulfoxide in 7fxw would not fit next to Arg127. Elimination of DMSO from a racemic mixture leads to an achiral indole-2-carboxylate that recruits a water molecule to bridge the ligand to Arg127. The two conformations of the chlorophenyl group were refined with 70:30 occupancy.

A possibly less obvious case of hydrolysis occurred in hFABP4 structure 7fxw, where a racemic mixture of a 3-keto-sulfoxide would not be able to bind to FABP4 due to space restrictions (Fig. 15 ▸). After the attack of a water molecule at the 3-keto group to form a tetrahedral intermediate, a tautomer of DMSO may be eliminated and the 5,6-dichloro-3-(2-chlorophenyl)-1*H*-indole-2-carboxylate is formed. This carboxylate binds to both Arg107 and Arg127. A water-mediated hydrogen-bonding network is present that includes the side chain of Ser53.

### 3.7. Different regioisomers

Compounds in this category could in principle be a result of mishaps during compound registration. Here, we assume their origin is due to unanticipated chemical side reactions.

#### 3.7.1. Shift of a methyl group in a pyrimidinone

**Figure 16:**
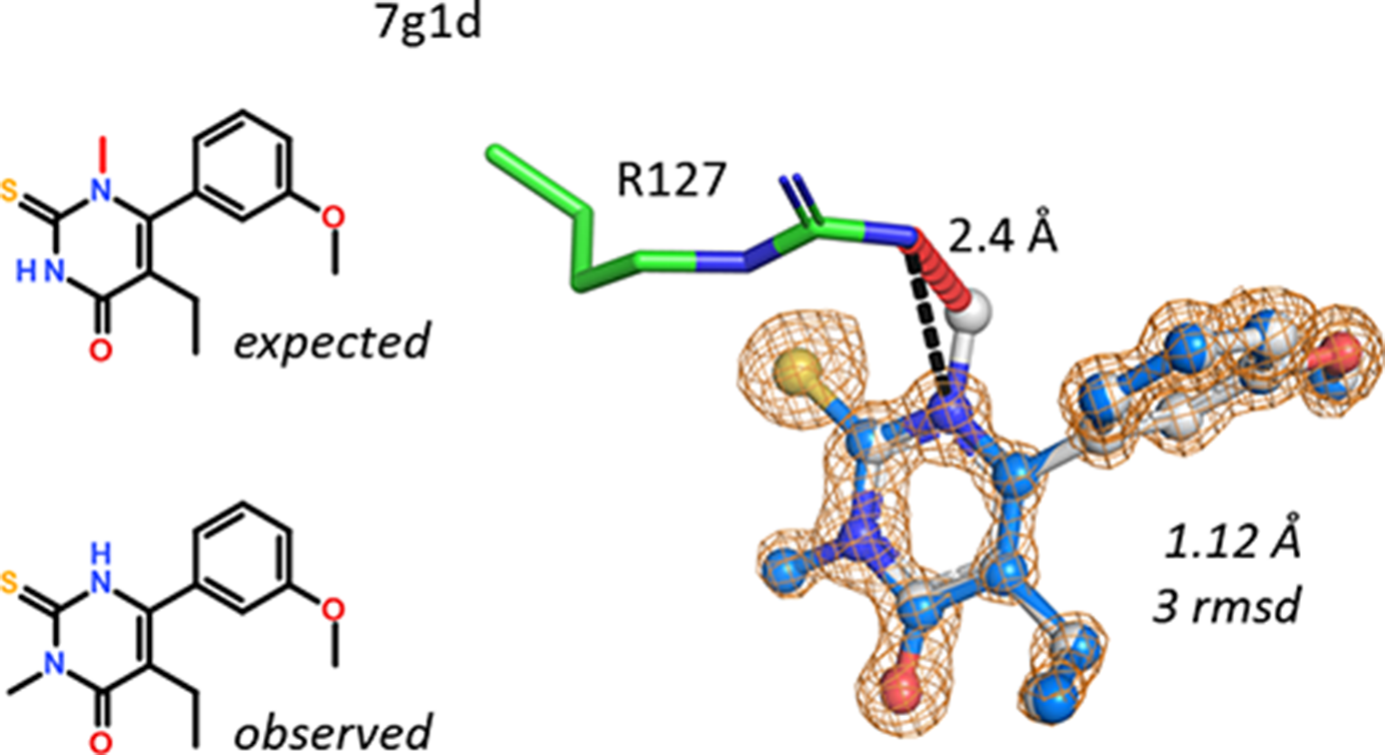
N–N methyl shift in a pyrimidinone. The methyl group, as expected, would clash with the guanidinium group of Arg127 (marked in red). The pyrimidinone cannot be rotated by 180°, as this would swap the positions of the exocyclic S and O atoms. The position of the S atom (yellow), however, is clear from the electron density.

The overall orientation of a 2-thioxo-pyrimidin-4-one ligand in hFABP4 structure 7g1d was clear from the strong electron density of the S atom. Alas, in this orientation the N-methyl group would clash at only 2.4 Å distance to the guanidinium group of the nearby Arg127, and there is extra electron density at the other N atom of the pyrimidinone (Fig. 16 ▸). Hence, a shift of the methyl group had to be assumed. As no other analytical data are available for this compound, the assignment is based solely on the 1.12 Å resolution map and stereochemical considerations.

#### 3.7.2. Shift of a methyl group between carboxylic acids

**Figure 17:**
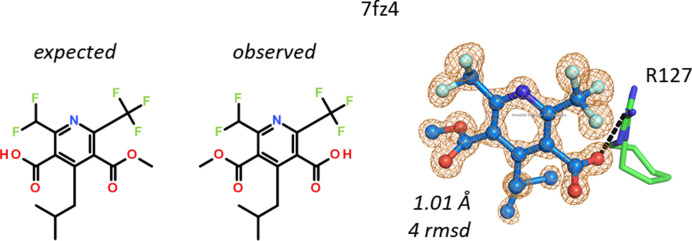
O–O methyl shift in a pyrimidinedicarboxylic acid. The methyl ester is located on the other carboxylic acid than expected. The ligand core is placed unambiguously due to the difference between the CF_3_ and CF_2_H groups.

In the pyrimidine-3,5-dicarboxylic acid ligand in structure 7fz4, one of the carboxylic acid groups is a methyl ester. The compound is almost symmetric, except that *ortho* to the carboxylic acids there is either a CF_3_ or a CF_2_H group. The difference of a single F atom allows unambiguous placement of the core of the ligand. After placement, the methyl group of the ester clashes with Arg127 and extra electron density is present at the other carboxylate group, necessitating revision of the structure of the ligand by swapping the ester to the other carboxylate (Fig. 17 ▸).

#### 3.7.3. Swap of hydroxyl and sulfhydryl groups

**Figure 18:**
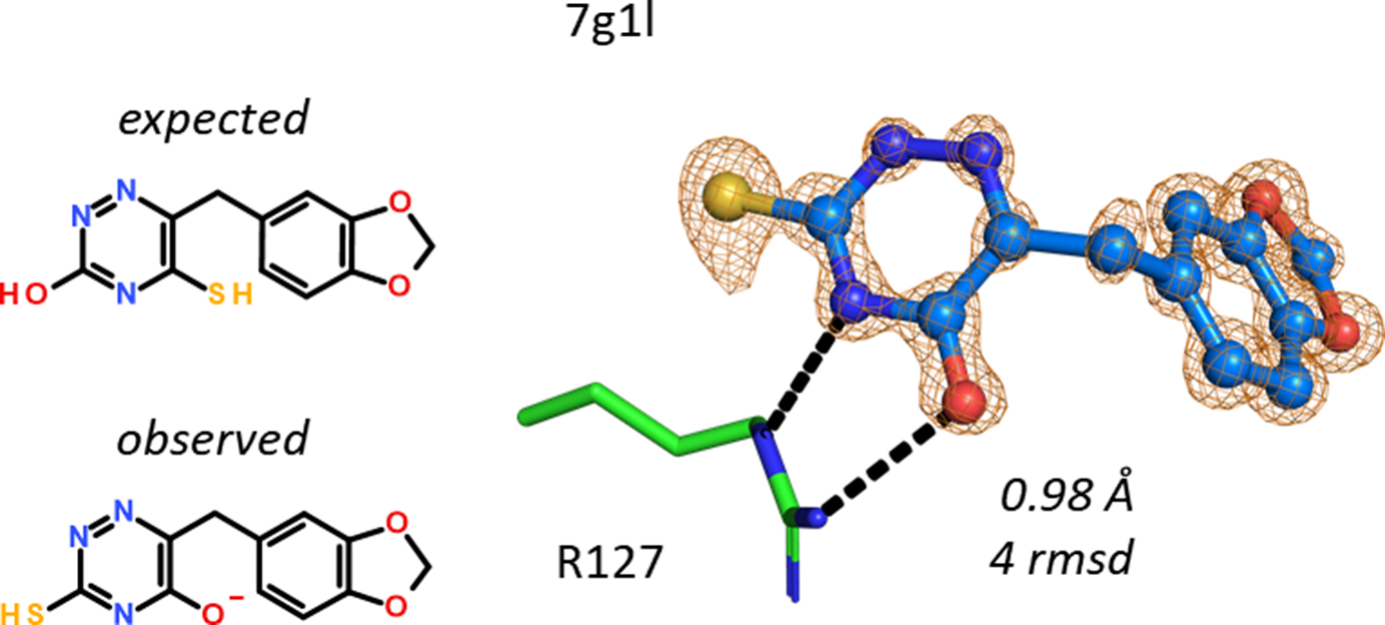
SH- and OH-group swap. The revised ligand identity is guided by the exit vector to the benzodioxole substituent and the strong electron density for the S atom. A bidentate hydrogen bond to the side chain of Arg127 is present.

The placement of a triazine compound containing both an SH and an OH group was guided by the strong electron density for the S atom in 7g1l but placed the benzodioxole substituent outside the omit electron density. Likewise, if the benzodioxole and triazine parts were fitted, the SH and OH groups are swapped, which is not supported by the strong electron density of the S atom. Thus, the two groups had to be reassigned by swapping in the revised compound structure (Fig. 18 ▸). The calculated p*K*_a_ value of the hydroxyl group in the ligand is 4.3, indicating that the enolate might have bound to the positively charged Arg127 of hFABP4.

#### 3.7.4. Shift of a single O atom in a pyran

**Figure 19:**
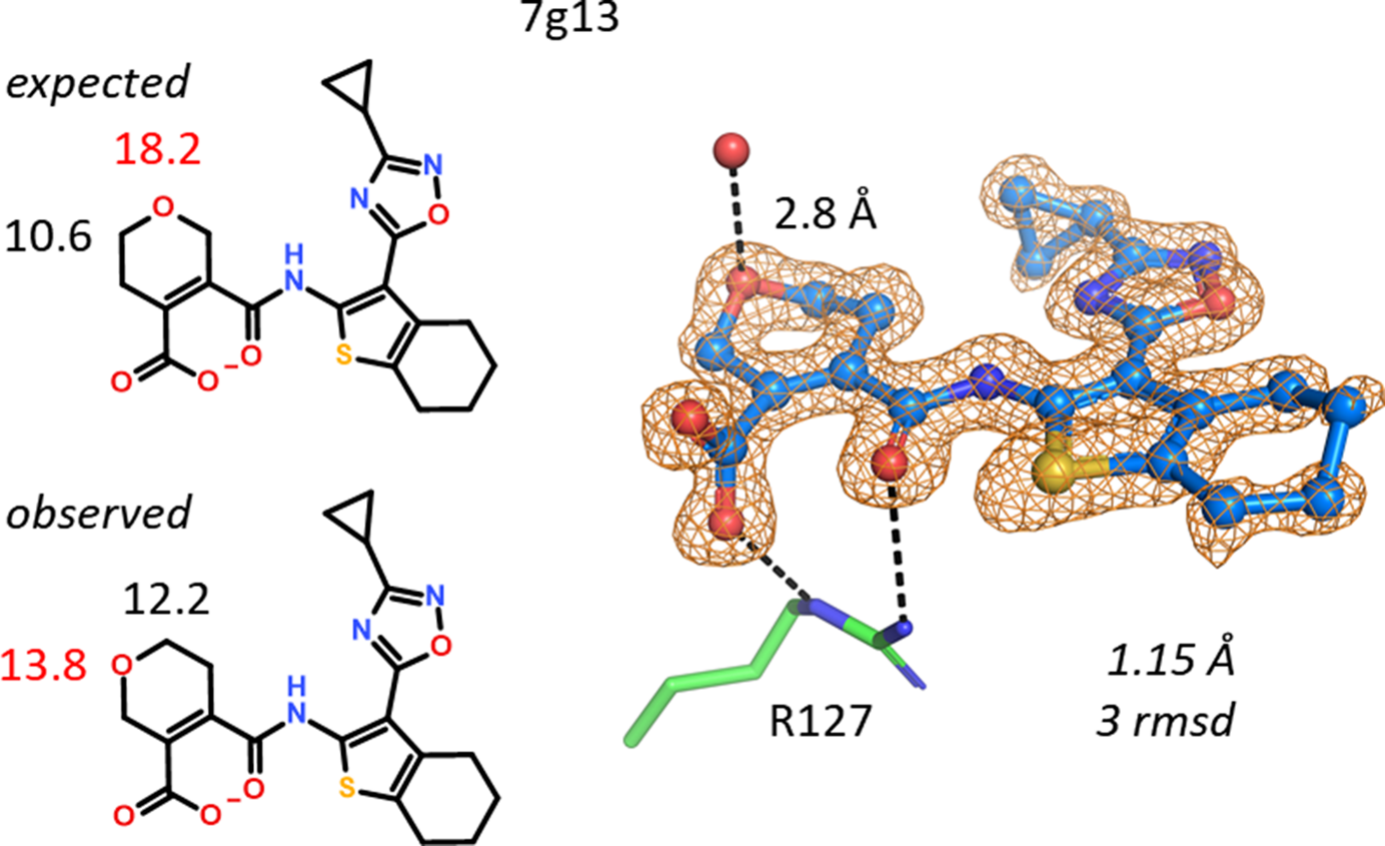
A different pyran. The shape of the electron density did not give an indication of an incorrect regioisomer. Instead, *B*-value refinement and a water contact (2.8 Å) identified the correct position of the pyran O atom. The *B* values after refinement of the incorrect and correct ligands are stated in units of Å^2^. Note the excessively large *B* value of 18.2 Å^2^ compared with the neighboring C atom (10.6 Å^2^) when oxygen is in the wrong position. ‘Swapping’ of the two atoms and refinement yields more similar *B* values of 13.8 and 12.2 Å^2^ for the O and C atoms, respectively.

A pyran-carboxylate ligand was soaked into a hFABP4_5 crystal and the expected structure of the ligand could be built into the 1.15 Å resolution omit map without immediately obvious problems. The cyclopropyl ring bound to the oxadiazole adopts a torsion angle suited to cover the lone electron pair of the *ortho* N atom, the thiophene S atom and the carbonyl group at 1,4-distance are in plane, and the pucker of the dihydropyran is, as expected, in a near-boat conformation. However, during refinement of 7g13 it was noticed that a water molecule is positioned close to a C atom in the pyran. Also, the *B* value of the O atom in the pyran refined to an abnormally high value when compared with the other atoms in the ring, indicating too many electrons at this position. Shifting the O atom to the position where it can hydrogen-bond to the water molecule followed by refinement reduced its *B* value to match those of nearby atoms (Fig. 19 ▸). In the absence of NMR data, the reassignment of the regio­isomer relies solely on *B*-value refinement and hydrogen-bonding criteria. In support, virtually the same situation is present in 7g07, where the same ligand is bound to hFABP4.

### 3.8. Addition reaction to thiophene

**Figure 20:**
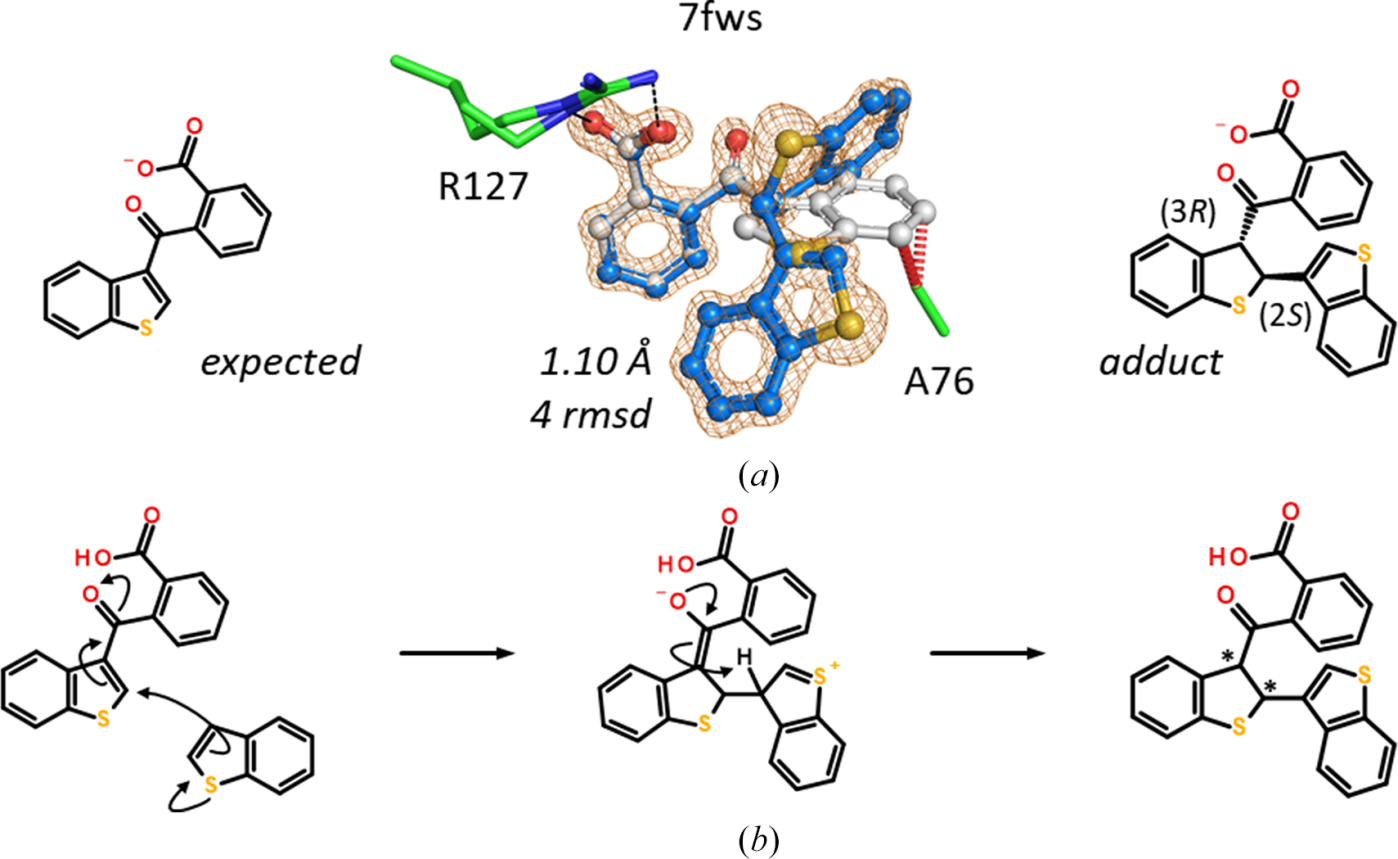
(*a*) 2-(1-Benzothiophene-3-carbonyl)benzoic acid (left) is too small to fit into the *F*_o_ − *F*_c_ omit map contoured at 4 r.m.s.d. (gray sticks) and would lead to clashes (red dashes). The extra density is fitted by an additional thiophene moiety added to the double bond of the first thiophene. The crystal selects a single diastereomer. (*b*) Proposed reaction scheme where thiophene acts as both a nucleophilic enol and an electrophilic 1,4 Michael system. The products have two chiral centers (asterisks).

The thiophene in the ligand bound to 7fvu, an 1.24 Å resolution hFABP4 structure, is too small to explain the omit electron-density map. Using the strong electron density of the S atoms in thiophene as a guide (Fig. 20 ▸), a second thiophene moiety covalently connected to the original ligand is present in the structure. While the mass of the original ligand was detected in the ligand solution, the UV purity of the sample was <80%, indicating the presence of additional components. Unfortunately, mass analysis did not cover the range necessary to detect the heavier adduct. The extra thiophene is probably a byproduct from the synthesis, but since the molecule was purchased from an external source, the synthesis route could not be traced back. Likely, excess thiophene in the reaction underwent an addition reaction to the 1,4-conjugated system of the target ligand, leading to the generation of two chiral centers. Since these addition reactions usually occur as *trans* additions, only two diastereomers are expected, of which the crystal selected the (2*S*,3*R*)-enantiomer, essentially acting as a ‘chiral sponge’ (see also Section 3.9).

Modeling of the original compound into the omit electron density shows severe clashes of the thiophene moiety with Ala76 of hFABP4. The kinked, more three-dimensional adduct fits into the binding pocket, with the second thiophene moiety entertaining van der Waals interactions with Phe17, Met20, Phe58 and Ala76.

### 3.9. FABP4 crystals as ‘chiral sponges’

With proteins being chiral, ligand-binding sites are chiral as well. The chirality of ligand-binding sites can be a powerful discriminator against different enantiomers and diastereo­mers, especially if the binding site is buried in the protein interior and has features imposing directionality such as hydrogen-bond donors and acceptors, as is the case with FABP.

#### 3.9.1. Racemate resolution

**Figure 21:**
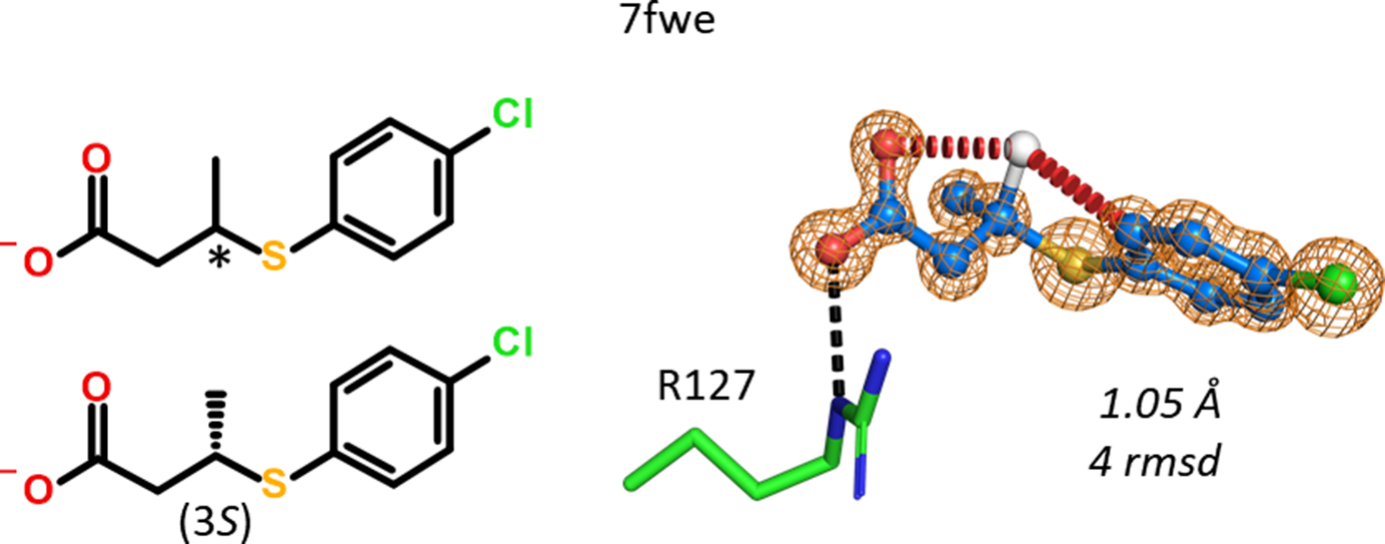
Selective binding of the 3*S*-enantiomer of 3-(4-chlorophenyl)sulfanyl­butanoic acid to hFABP4. The (3*R*)-enantiomer is shown in white as a reference with the intramolecular clashes (red dashes; 2.6–2.8 Å) that it would suffer in this ligand conformation.

A racemic mixture of 3-(4-chlorophenyl)sulfanylbutanoic acid was soaked into hFABP4 and data were collected to 1.05 Å resolution. Only the (3*S*)-enantiomer, in which the methyl group points away from both the carboxylate and chlorophenyl moieties, was bound. Modeling of the (3*R*)-enantiomer is possible from the standpoint of the protein: there is ample space in the binding site filled by water molecules that might be displaced by ligand atoms. However, in the (3*R*)-enantiomer the methyl group comes close to both the carboxylate and chlorophenyl moieties, prohibiting a binding-competent ligand conformation (Fig. 21 ▸).

Selective binding of enantiomers by proteins has implications for IC_50_ and *K*_d_ measurements performed with racemic mixtures. If the affinity of the enantiomers is similar, the IC_50_ values for the racemate will represent those for the enantiomers. By contrast, if only a single enantiomer binds, the concentrations used for the titration are off by a factor of two.

#### 3.9.2. Assignment of the correct chirality to the products of a racemic separation

**Figure 22:**
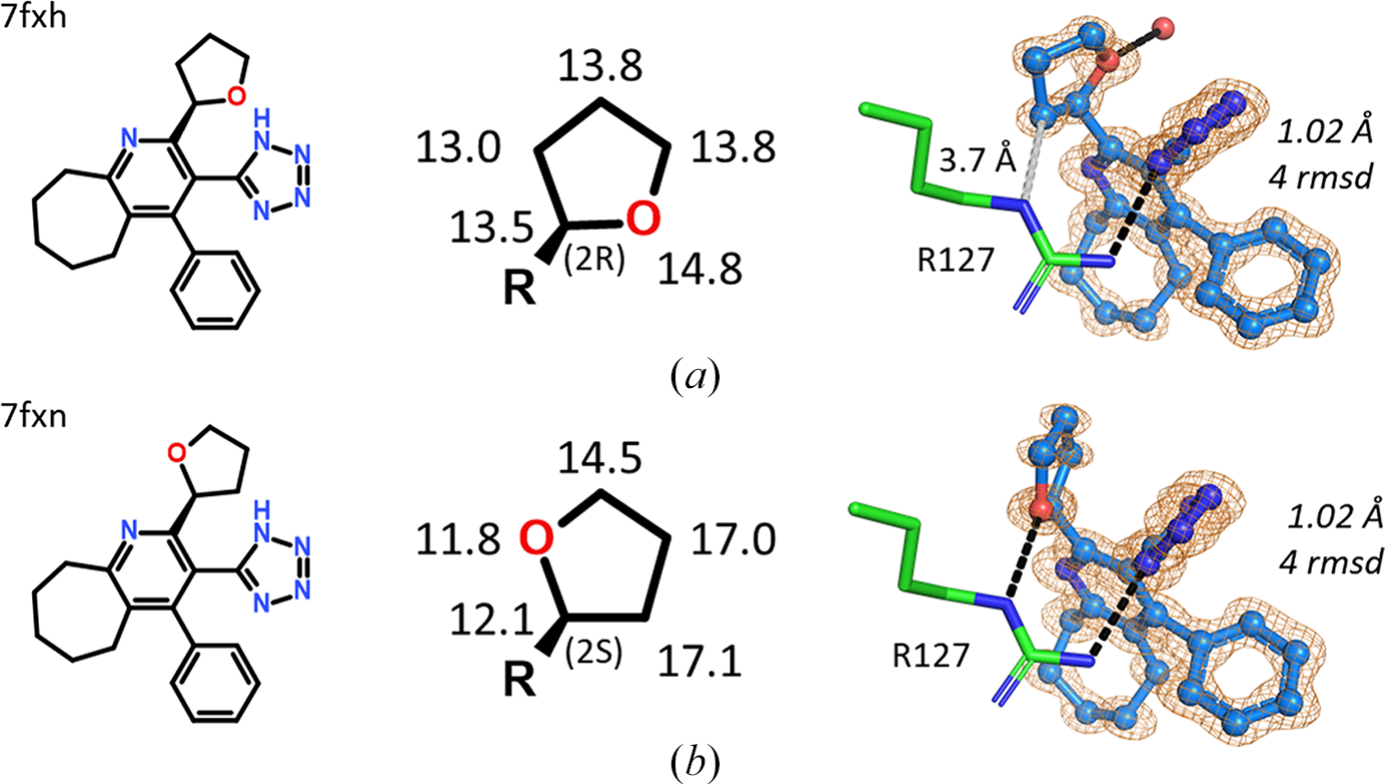
Structures of individual enantiomers in complex with hFABP4. Both cases had 1.02 Å resolution and the omit maps are contoured at 4 r.m.s.d. The calculated p*K*_a_ value for both enantiomers is 3.8. The anisotropically refined *B* values for the oxolane moiety in the structures are given in units of Å^2^ in the middle panel (*R* is the remainder of the ligand). The final assigned chirality at the oxolane is also indicated as (2*R*) for 7fxh and (2*S*) for 7fxn. (*a*) The van der Waals contact between the C atom and Arg127 in 7fxh is 3.7 Å (dashed gray line). The O atom points away from Arg127 and recruits a water molecule. (*b*) In the other enantiomer, the O atom makes direct interactions with Arg127.

Tetrazoles were among the most promising almost isosteric replacements for the fatty-acid carboxylate group (Kuhne *et al.*, 2016 ▸). Their calculated p*K*_a_ values are about 3.8, in the same range as those for fatty acids. The racemic mixture of a tetrazole carrying a chiral oxolane (tetrahydrofuran) group was resolved and X-ray crystallography was used to assign the correct enantiomers (Fig. 22 ▸). The problem can be solved purely by geometric considerations when analyzing the interactions of the oxolane moieties in hFABP4 structures 7fxh and 7fxn. In 7fxh the O atom of the oxolane points away from the nearby guanidinium group of Arg127, recruiting a water molecule. The C atom of the oxolane closest to Arg127 is at van der Waals distance (3.7 Å). By contrast, in 7fxn the O atom of the oxolane hydrogen-bonds to the Arg127 guanidinium group, enabled by a slight rotation of the oxolane about the bond to the pyridine core that brings the hydrogen-bonding partners into close proximity. All other ligand interactions are virtually identical when 7fxh and 7fxn are compared. Unfortunately, the IC_50_ values of the individual enantiomers were not measured.

Independent evidence for the correct assignment of the enantiomers comes from anisotropic *B*-value refinement (middle panel of Fig. 22 ▸). In 7fxh, the distribution of *B* values within the oxolane is rather homogeneous, indicating correct element assignment. The ratio of the *B* values of the oxygen and its opposing carbon is 1.14. If the O atom and its opposing C atom are swapped, *i.e.* the wrong enantiomer is built, the ratio of the refined *B* values is 18.1/12.1 Å^2^ = 1.50, showing that the *B* value of the O atom in this position is too large and that of the C atom is too small, in agreement with the larger number of electrons in oxygen compared with carbon. The case is less clear-cut in 7fxn, where the *B*-value distribution in the oxolane is wider than in 7fxh, likely owing to pucker flexibility of the oxolane and the fixing of the O atom in a hydrogen bond to Arg127, which may reduce its *B* value. Nevertheless, the smallest *B* value is that of the assigned O atom (11.8 Å^2^) and refinement of the wrong enantiomer gives an unusually large *B* value for the O atom (16.8 Å^2^) and a too small a *B* value for the C atom (9.8 Å^2^).

### 3.10. Identification of unexpected ligands at lower resolution

The case of FABP described here is exceptional due to the high resolution of most of the structures, which greatly facilitated the identification of ligands of unexpected composition. In addition, the high tolerance of the crystals for DMSO allowed soaking with high final ligand concentrations of 60 m*M*, and most ligands were soluble under the crystallization conditions plus the 30% DMSO from the ligand solution. Thus, impurities that might not have been detected under less favorable circumstances may have materialized in FABPs, skewing the data set to an unusually large number of unexpected outcomes.

To detect ‘wrong’ ligands at worse resolutions, those ligands need to be either very well defined by electron density or modified strongly compared with the expected molecule. ‘Strongly’ in this context means either a significant change in three-dimensional shape that cannot be explained by a conformation, or an addition of sufficient molecular mass that is ordered in the crystal structure. A loss of mass may go unnoticed when that part of the ligand is solvent-exposed and hence flexible in the crystal. We have recently released ∼200 crystal structures each of the human phosphodiesterase 10 (PDE10) domain (Tosstorff *et al.*, 2022 ▸) and rat autotaxin (ATX; Hunziker *et al.*, 2021 ▸; Martin *et al.*, 2025 ▸) at resolution ranges between 1.5 and 2.5 Å. The number of unexpected ligands that could be clearly identified is much smaller in these data sets compared with the FABP data set, probably due to the lower mean resolution and the solvent-exposed active sites in PDE10 and ATX. Two examples each for PDE10 and ATX are reported here that show strongly modified ligands and that hence were readily identified from electron-density maps.

#### 3.10.1. PDE10

**Figure 23:**
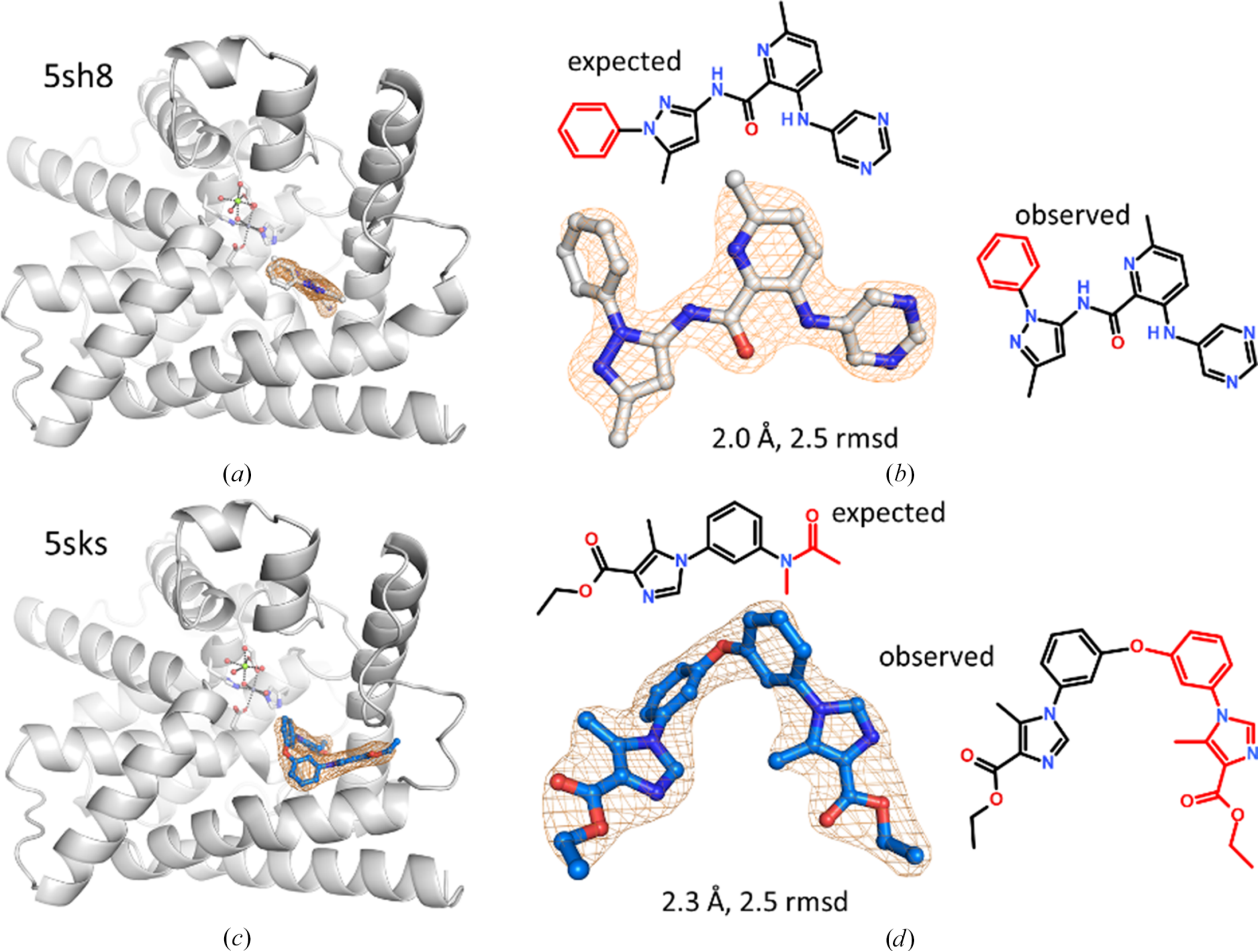
Unexpected ligands in PDE10. (*a*) Phosphodiesterase domain of human PDE10 in complex with an aminopyridine ligand. (*b*) The phenyl substituent of the pyrazole in 5sh8 is at the other N atom compared with the anticipated structure. (*c*) An aryl imidazole ligand binds at a similar site in 5sks. (*d*) This ligand has ‘dimerized’ by losing an *N*-methylacetyl group and forming a biphenyl ether.

PDE10 hydrolyzes the second messenger cGMP and is a potential target for treating schizophrenia. Its nuclease domain contains a catalytic binuclear Mg^2+^–Zn^2+^ complex in the active site with a nearby binding site for the nucleotide that can be blocked by inhibitors. The phenyl substituent at a pyrazole in 5sh8 was found at the other nitrogen and this isomer was confirmed by NMR. The phenyl pyrazole points towards bulk solvent and the phenyl group is not as well ordered as the rest of the ligand (Fig. 23 ▸*b*), but its position is clear. Interestingly, there are no clashes with PDE10 if the expected molecule is modeled at this position, so the synthesis might have led to the preferred isomer with the phenyl and methyl groups of the ligand in *meta* rather than *ortho* positions. A mixture of the two regioisomers is excluded by NMR and the lack of electron density at the nitrogen *ortho* to the methyl group.

An even more drastic change in chemical structure is observed in a phenyl imidazole ligand that should have carried an N-methyl acetyl group at the phenyl moiety but has ‘dimerized’ *via* an ether bridge into a biphenyl ether (Fig. 23 ▸*c*). Its presence as an impurity was confirmed by mass spectrometry and its origin might be from a metal catalyst used in the synthesis of the anticipated molecule. The hydrogen-bond acceptor at the newly introduced imidazole interacts with the side chain of Tyr693, but at the cost of low ligand efficiency, so this compound was not followed up by synthesis of derivatives. However, Tyr693 is a frequent hydrogen-bonding partner in other PDE10–ligand complexes.

#### 3.10.2. ATX

**Figure 24:**
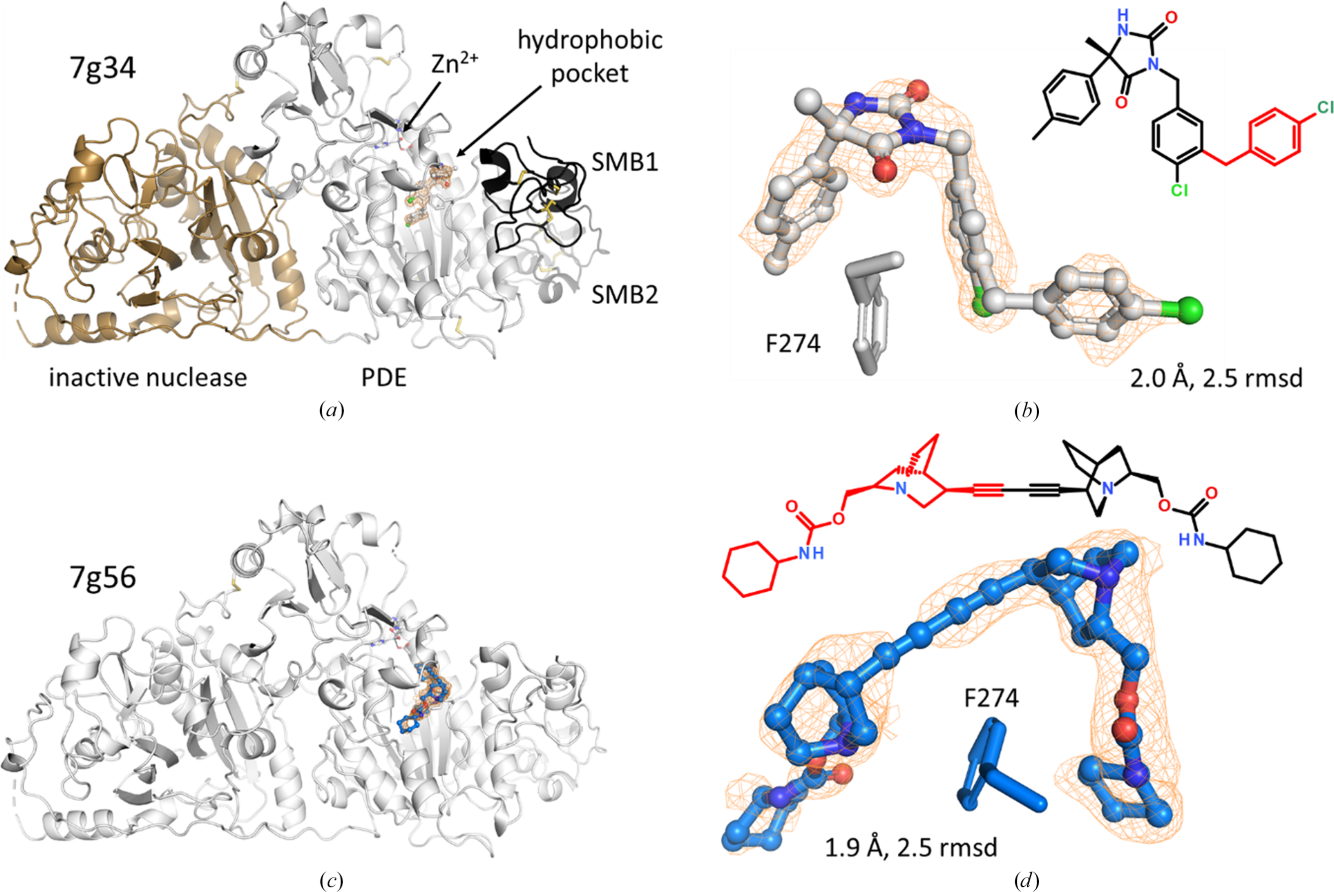
Unexpected ligands in autotaxin. (*a*) Domain organization in autotaxin. The two SMB domains are shown on the right in black and dark gray, the central PDE domain with the active-site Zn^2+^ in white and the inactive nuclease domain in brown to the left. (*b*) An additional 4-chlorotosyl group (red in the formula) is present in the hydantoin inhibitor (7g34), as detected both by an omit map contoured at 2.5 r.m.s.d. and as an impurity by mass spectrometry. (*c*) In 7g56, an inhibitor containing an acetylene group binds at the same site as the hydantoin inhibitor. (*d*) The acetylene inhibitor is dimerized by oxidation, *i.e.* it now has two triple bonds connected by a single bond. The duplicated part is shown in red in the formula. The dimer accounts for ∼90% of the total ligand and thus is the main product of the synthesis.

ATX also has phosphodiesterase activity, but its biological function is that of a Zn^2+^-dependent phospholipase, hydrolyzing lysophosphatidyl choline into the signaling molecule lysophosphatidic acid that stimulates cell proliferation. ATX is a target for treating some forms of cancer and for idiopathic pulmonary fibrosis (Hunziker *et al.*, 2021 ▸; Martin *et al.*, 2025 ▸). Its structure contains two N-terminal somatomedin-B-like (SMB) domains, a central PDE domain and a C-terminal inactive nuclease domain (Figs. 24 ▸*a* and 24 ▸*b*). In the example of a hydantoin derivative, the inhibitor detected by 1.9 Å resolution electron density and by mass spectrometry has an additional 4-chlorotosyl group that likely arises as a byproduct from the synthesis. The additional 4-chlorotosyl group points into a hydrophobic pocket and interacts with Ile167, Ala217, Phe273 and Phe274. The additional group appears to be flexible according to the lower electron density and slightly elevated *B* values compared with the rest of the ligand. While from these data it cannot be excluded that a mixture of the intended and modified hydantoin compounds has bound, the additional group may certainly inspire further design in this pocket.

In the second ATX example (Figs. 24 ▸*c* and 24 ▸*d*), an inhibitor with a terminal acetylene group exhibits electron density consistent with a dimer. According to NMR, 90% of this compound has undergone oxidation at the acetylene C—H bond to the dimer, so in this case the anticipated compound is a byproduct of the reaction and the dimer is the main product. The ‘second’ half of the inhibitor wraps around Phe274 and points towards bulk solvent, which may explain why this half is less well ordered and has lower electron density compared with the buried half of the ligand. Both inhibitors were soaked as racemic (7g34) and diastereomeric (7g56) mixtures, with the protein selecting a single enantiomer/diastereomer from the mixture.

## 4. Conclusions and outlook

Reports on mismatching ligands and electron density are scarce (Muller, 2013 ▸; Masmaliyeva *et al.*, 2020 ▸), with only a few reviews on the topic (Deller & Rupp, 2015 ▸; Ibrahim *et al.*, 2017 ▸; Wlodawer *et al.*, 2018 ▸) and web resources to test the quality of ligands (Pozharski *et al.*, 2013 ▸; Weichenberger *et al.*, 2013 ▸; Kowiel *et al.*, 2019 ▸). Since, in general, negative results tend not to be published (Nair, 2019 ▸), we can only assume that other structural biologists will have had similar experiences of unexpected chemical structures in their protein–ligand complexes. The case of FABP described here is somewhat special because the crystals allowed soaking with very high DMSO, and therefore ligand concentrations, possibly permitting the accumulation of impurities in the crystal, and the crystals still diffracted to very high resolution. For lower resolution structures such as the PDE10 and ATX examples described here, ligand modifications need to be much larger with respect to size and/or shape to be identified easily from the electron-density maps. A future study looking at the resolution-dependence of ligand omit map interpretability by truncating high-resolution data and adding random noise may provide quantitative criteria for when a ligand may be considered to be ‘truly identified’. This task seems especially important for interpreting the results of fragment or cocktail screens, where the electron-density shape can sometimes accommodate several ligand poses and the purity of the fragments may be unknown. Also, if hits from ‘direct to biology’ approaches, where crude reaction mixtures are submitted for a biological test to find potential active compounds, are followed up by X-ray crystallography, ligand ambiguities may arise.

If the percentage of misassigned ligands in the FABP set is only slightly predictive of any other set of ligand-bound structures, there is a chance of a fair number of misassignments, particularly in structures determined at resolutions that do not allow the resolution of individual atoms and when the chemical change in the ligand is small compared with expectation. It has been noted that 20–25% of ligand-containing crystal structures are likely to be incorrectly refined (Liebeschuetz *et al.*, 2012 ▸) and that about 12% of protein–ligand crystal structures in the PDB are just ‘bad’ (Deller & Rupp, 2015 ▸). Even honest errors in ligand chemistry and ligand modeling that have gone unnoticed in the absence of expectation/confirmation bias on the part of the structural biologist impart a negative effect on the quality of the training set used for those ML algorithms that predict binding poses and/or relative affinities, unless the problematic structures are removed from the training set, for example by filtering with ligand-centered real-space correlation coefficients (RSCCs; Brändén & Jones, 1990 ▸). As a basis for discussion among structural biologists, a training set from crystal structures could contain only those structures that have associated diffraction data for map-reproduction purposes and meet a number of global and local requirements. The diffraction data should not display any pathologies that change intensities in a systematic way to reduce the signal-to-noise ratio, such as twinning, pseudo-translation or anisotropy, although the absence of anisotropy seems next to impossible. Among the global criteria, a minimum high-resolution limit of maybe 2–2.5 Å in all reciprocal directions and an expected *R*_free_ < 24% according to the Tickle formula might be appropriate (Tickle, 2012 ▸). Then, after (automatic?) refinement of the ligand-containing structure with the current best protocols at full ligand occupancy and carefully prepared restraints, local criteria need to be met. These may include no alternate conformations in both the ligand and the ligand-binding sites, no *B* values of side-chain and ligand atoms significantly (twofold?) higher than the average of the atoms within and lining the binding site, and a high RSCC of the ligand. A per-atom RSCC calculated against the 2*F*_o_ − *F*_c_ map for the ligand after refinement could be used to judge whether it is ‘present’ with all atoms. If just a single ligand atom is present with RSCC < 0.9, the structure should be disregarded or maybe used with a flag indicating its reduced reliability. This procedure probably leaves very few structures because it would remove all otherwise good ligand complexes with rotationally disordered substituents. The training set would be small but reliable. We have not performed this calculation because the quality criteria should first be agreed upon by the community.

In addition, the number of well refined ligand-containing crystal structures could be increased. We therefore encourage colleagues from the pharmaceutical industry to deposit their unpublished, high-quality, ligand-bound legacy structures plus some affinity data with the PDB so that future prediction algorithms may benefit from high-quality and diverse training sets. We are aware that the time needed to deposit many structures in a single session is often prohibitively long, despite helpful tools such as PDB group deposition (https://deposit-group.rcsb.rutgers.edu/groupdeposit) and *GEMMI* (https://gemmi.readthedocs.io). The very human urge to re-refine older models with the latest software to update them to current standards adds to the time requirements, although this task could be delegated to *PDB-REDO* (Joosten *et al.*, 2014 ▸). Collecting the information that is necessary or just beneficial for a ‘proper’ deposition, including X-ray scaling statistics, restraints, SMILES, affinity data *etc.*, may also be considered too time-consuming, especially for very old (>15 years) structures for which this information may be difficult to retrieve or may not even exist any more. Our hope is that in the near future the final refinement, preparation and deposition of legacy structures can be automated with minimal human intervention so as to obtain a large, diverse and reliable set of ligand-bound crystal structures that form a solid base for ML.

## Supplementary Material

Supplementary Excel File S1 detailing the crystal structures, ligands and IC50 values. DOI: 10.1107/S2059798325006096/gm5113sup1.xlsx
